# Low-Molecular-Weight Chitosan Produced by Gamma Irradiation and Hydrogen Peroxide Promotes Immune Readiness, Antioxidant Responses, and Survival in *Photobacterium damselae*-Challenged Asian Seabass (*Lates calcarifer*)

**DOI:** 10.3390/ijms27177772

**Published:** 2026-08-30

**Authors:** Thitirat Rattanawongwiboon, Natthapong Paankhao, Ratchanon Chumchuen, Nattaset Chinnabutra, Wasin Saisin, Benchawan Kumwan, Pakapon Meachasompop, Yosapon Adisornprasert, Pimrawee Chaemlek, Prapansak Srisapoome, Passakorn Kingwascharapong, Theeranan Tangthong, Wararut Buncharoen, Anurak Uchuwittayakul

**Affiliations:** 1 Thailand Institute of Nuclear Technology (Public Organization), Nakhon Nayok 26120, Thailand; thitirat@tint.or.th (T.R.); theeranan@tint.or.th (T.T.); 2 Kamphaeng Saen Fisheries Research Station, Faculty of Fisheries, Kasetsart University, Kamphaeng Saen Campus, Nakhon Pathom 73140, Thailand; ffisnpp@ku.ac.th; 3 Department of Aquaculture, Faculty of Fisheries, Kasetsart University, Bangkok 10900, Thailand; ratchanon.chum@ku.th (R.C.); nattaset.c@ku.th (N.C.); wasin.sai@ku.th (W.S.); benchawan.kumw@ku.th (B.K.); pakapon.meac@ku.th (P.M.); yosapon.ad@ku.th (Y.A.); pimrawee.ch@ku.th (P.C.); ffispssp@ku.ac.th (P.S.); 4 Laboratory of Aquatic Animal Health Management, Department of Aquaculture, Faculty of Fisheries, Kasetsart University, Bangkok 10900, Thailand; 5 Center of Excellence in Aquatic Animal Health Management, Faculty of Fisheries, Kasetsart University, Bangkok 10900, Thailand; 6 Department of Fishery Products, Faculty of Fisheries, Kasetsart University, Bangkok 10900, Thailand; passakorn.ki@ku.th; 7 Department of Biology, Faculty of Science, Chiang Mai University, Chiang Mai 50200, Thailand; wararut.bun@cmu.ac.th

**Keywords:** Asian seabass, *Lates calcarifer*, low-molecular-weight chitosan, gamma irradiation, hydrogen peroxide depolymerization, antioxidant defense, immune response, mucosal immunity, *Photobacterium damselae*, disease resistance

## Abstract

Low-molecular-weight chitosan may exhibit improved dispersion and biological accessibility compared with high-molecular-weight chitosan, but direct comparisons in Asian seabass remain limited. This study evaluated dietary low-molecular-weight chitosan produced by combined gamma irradiation and hydrogen peroxide depolymerization. The untreated and degraded preparations had apparent GPC-derived molecular weights of approximately 85 and 10 kDa, respectively. Juvenile Asian seabass (*Lates calcarifer*) were fed a Control diet of 1.0% high-molecular-weight chitosan or low-molecular-weight chitosan at 0.25%, 0.5%, or 1.0% for four weeks. Growth, serum antioxidant and humoral immune parameters, tissue-specific gene expression, and resistance to *Photobacterium damselae* were evaluated. No treatment significantly affected growth or feed utilization. Both high- and low-molecular-weight chitosan reduced serum MDA relative to the Control, whereas selected antioxidant and immune responses differed among inclusion levels. The 0.5% low-molecular-weight treatment increased GSH and GPx activity, the 1.0% low-molecular-weight treatment produced the highest CAT activity, and the 0.25% low-molecular-weight treatment produced the highest total serum IgM. Low-molecular-weight chitosan generally produced broader tissue-specific transcriptional responses than 1.0% high-molecular-weight chitosan. Following bacterial challenge, all chitosan treatments improved survival relative to the Control, with the highest numerical RPS observed in the 0.5% low-molecular-weight group. These results indicate that molecular-weight reduction influenced the biological response to dietary chitosan. Among the tested levels, 0.5% produced the most consistent combined response, although the optimal level was endpoint-specific and requires longer-term validation.

## 1. Introduction

Asian seabass (*Lates calcarifer*) is one of the most important carnivorous fish species cultured in tropical and subtropical aquaculture systems. Its rapid growth, high market value, broad salinity tolerance, and suitability for both freshwater and brackish-water farming have contributed to its increasing production in many Asian countries [1]. However, the intensification of seabass farming has been accompanied by frequent disease outbreaks, environmental stress, and reduced health performance, particularly during nursery and grow-out stages [2,3]. Bacterial diseases caused by opportunistic pathogens remain a major constraint to sustainable seabass production, leading to reduced feed intake, impaired growth, increased mortality, and substantial economic losses [4].

*Photobacterium damselae* is a halophilic Gram-negative pathogen associated with photobacteriosis and systemic disease in cultured marine fish. In Asian seabass, *P. damselae* subsp. *damselae* has been isolated from naturally diseased fish exhibiting external and internal pathological signs, and experimental infection has confirmed its virulence in this species [5]. More recently, pathogenic isolates recovered from commercial Asian seabass farms in Thailand were characterized as *P. damselae* subsp. *damselae*, further demonstrating the continuing relevance of this pathogen to seabass aquaculture [6]. The pathogen possesses multiple virulence-associated mechanisms, including hemolytic and cytotoxic activities, which can contribute to tissue injury, systemic infection, and mortality [7]. Moreover, antimicrobial resistance detected among isolates from farmed Asian seabass highlights the need for preventive approaches that strengthen host defenses and reduce dependence on therapeutic antibiotics [6].

The control of bacterial diseases in aquaculture has historically relied on antibiotics and chemical treatments. However, the continued use of these agents has raised concerns regarding antimicrobial resistance, environmental contamination, drug residues, and consumer safety [8,9,10,11]. Therefore, the development of safe and sustainable nutritional strategies that enhance fish immunity and disease resistance has become increasingly important. Functional feed additives derived from natural biopolymers are of particular interest because they can support health performance while reducing dependence on antibiotics [9,10].

Chitosan, a deacetylated derivative of chitin, is a biodegradable, biocompatible, and nontoxic biopolymer commonly obtained from crustacean shell waste [4,12]. It possesses several biological properties relevant to aquaculture, including antimicrobial, antioxidant, immunomodulatory, and film-forming activities. These characteristics make chitosan a promising candidate for functional aquafeeds. Nevertheless, the biological activity of chitosan is strongly influenced by its molecular weight, solubility, viscosity, and degree of interaction with biological surfaces [13,14,15]. High-molecular-weight chitosan exhibits useful structural properties but often has limited solubility and lower biological availability. In contrast, low-molecular-weight chitosan generally shows improved solubility, lower viscosity, better dispersion, and stronger interaction with mucosal surfaces, which may enhance its biological efficacy in fish [16,17].

Synergistic degradation using gamma irradiation in combination with hydrogen peroxide is an effective approach for producing low-molecular-weight chitosan. Gamma irradiation induces polymer chain scission, while hydrogen peroxide promotes the generation of reactive hydroxyl radicals that accelerate depolymerization. This combined process can reduce molecular weight efficiently while preserving key functional groups associated with chitosan bioactivity. As a result, synergistically degraded low-molecular-weight chitosan may offer improved functional properties compared with native chitosan and may be effective at lower dietary inclusion levels [18].

Previous studies have shown that dietary chitosan, chitosan oligosaccharides, and chitosan nanoparticles can influence growth, antioxidant status, innate immunity, intestinal health, and disease resistance in several aquatic species, including Nile tilapia, Asian seabass, and hybrid catfish [17,19,20,21,22]. However, the responses vary with molecular weight, degree of deacetylation, dose, species, and feeding duration. Direct dietary comparisons between native chitosan and low-molecular-weight chitosan remain limited, particularly in Asian seabass. Moreover, most previous aquaculture studies have used commercially available chitosan, enzymatically produced oligosaccharides, or nanoparticle formulations rather than material produced through combined gamma irradiation and hydrogen peroxide depolymerization [17]. The combined process offers several potential advantages. Gamma irradiation produces polymer-chain scission without requiring a chemical initiator, while hydrogen peroxide generates reactive species that accelerate depolymerization. Their combined action can achieve substantial molecular-weight reduction at a lower irradiation dose or shorter treatment duration than irradiation or oxidative treatment alone [18]. The process also produces a water-dispersible low-molecular-weight material without requiring a separate nanoparticle carrier. Nevertheless, the biological performance of the resulting material must be evaluated empirically because its effects can vary among fish species and physiological endpoints.

Comparative evidence indicates that molecular weight is an important determinant of chitosan activity. In black tiger shrimp, chitin, chitosan, chitosan oligosaccharide, and N-acetyl-D-glucosamine produced different growth and antioxidant responses [23]. In blunt snout bream head-kidney macrophages, chitosan oligosaccharides of different molecular weights induced distinct immunomodulatory responses [24]. These findings suggest that lower molecular weight does not simply increase all biological effects uniformly; rather, it may alter solubility, cellular interactions, and endpoint-specific activity. Direct in vivo comparisons of native and degraded chitosan using equivalent or overlapping dietary levels remain limited in fish, supporting the need for the present evaluation in Asian seabass.

Therefore, this study directly compared native high-molecular-weight chitosan with low-molecular-weight chitosan produced by combined gamma irradiation and hydrogen peroxide treatment. Their dietary effects on growth, feed utilization, serum antioxidant and humoral immune parameters, tissue-specific transcriptional responses, and resistance to *Photobacterium damselae* were evaluated in Asian seabass. The integration of physicochemical characterization with systemic, mucosal, and post-challenge endpoints provides a species-specific assessment of whether molecular-weight reduction improves the functional performance of dietary chitosan.

## 2. Results

### 2.1. Growth Performance and Feed Utilization

**Figure 1:**
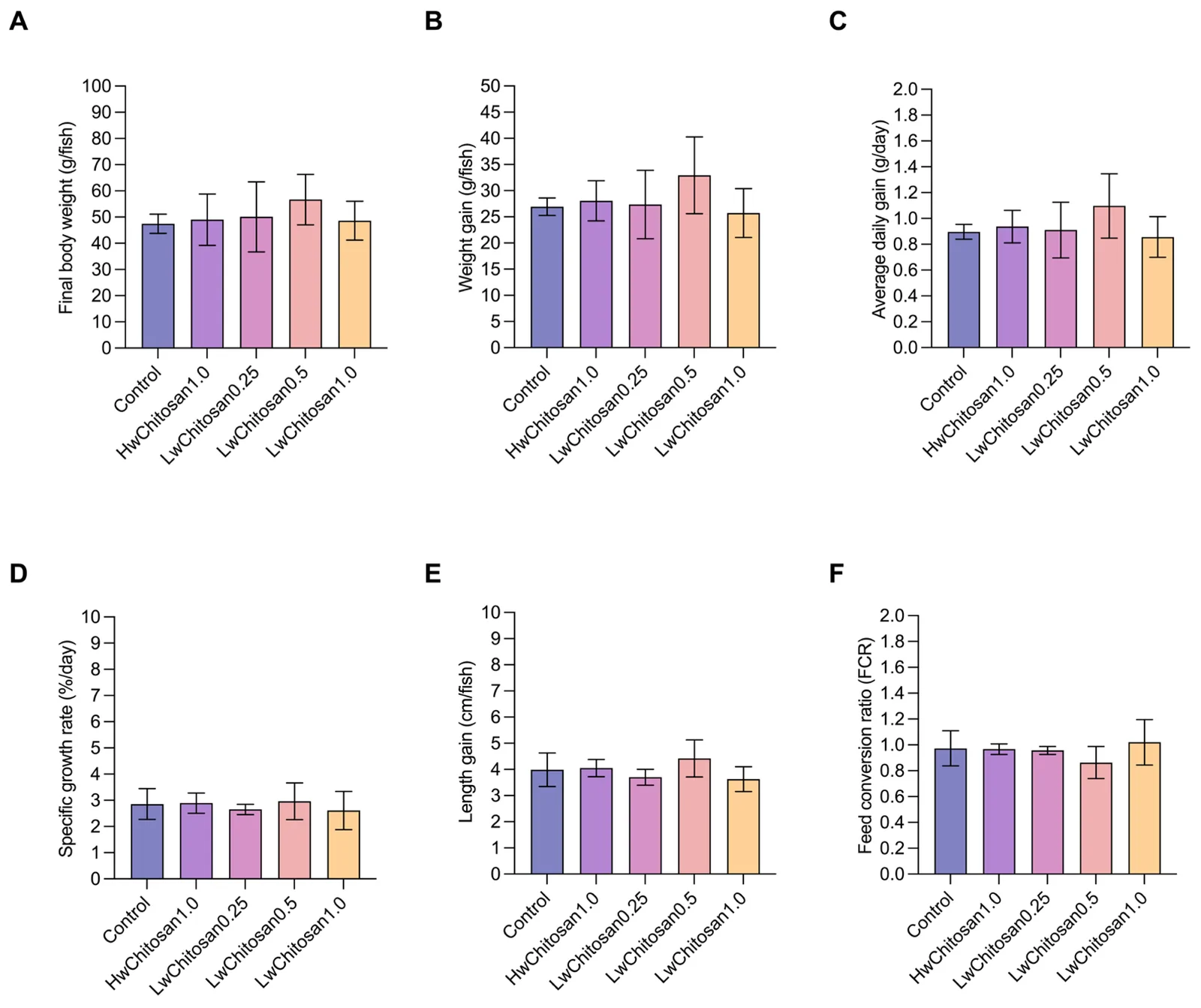
Growth performance and feed utilization of Asian seabass (*Lates calcarifer*) fed experimental diets for 4 weeks. (**A**) Final body weight, (**B**) weight gain, (**C**) average daily gain, (**D**) specific growth rate, (**E**) length gain, and (**F**) feed conversion ratio. Experimental diets included Control, HwChitosan1.0, LwChitosan0.25, LwChitosan0.5, and LwChitosan1.0. Values are presented as mean ± SEM with *n* = 4 tanks per treatment. No significant differences were observed among treatments (*p* > 0.05).

The effects of dietary chitosan supplementation on the growth performance and feed utilization of Asian seabass are presented in Figure 1A–F. After the 4-week feeding trial, dietary treatment did not significantly affect final body weight, weight gain, average daily gain, specific growth rate, length gain, or feed conversion ratio (one-way ANOVA, *p* > 0.05).

Final body weight ranged from 47.46 ± 3.69 to 56.68 ± 9.63 g/fish and did not differ significantly among treatments (*p* = 0.765). Fish fed the LwChitosan0.5 diet had the highest numerical final body weight at 56.68 ± 9.63 g/fish, followed by the LwChitosan0.25, HwChitosan1.0, LwChitosan1.0, and Control groups, with values of 50.09 ± 13.39, 49.00 ± 9.77, 48.61 ± 7.42, and 47.46 ± 3.69 g/fish, respectively.

Weight gain was also not significantly affected by dietary treatment (*p* = 0.524). The highest numerical weight gain was recorded in the LwChitosan0.5 group at 32.94 ± 7.34 g/fish, whereas the HwChitosan1.0, LwChitosan0.25, Control, and LwChitosan1.0 groups showed weight gains of 28.07 ± 3.84, 27.35 ± 6.54, 26.93 ± 1.68, and 25.73 ± 4.66 g/fish, respectively.

Similarly, average daily gain did not differ significantly among treatments (*p* = 0.530). Fish fed LwChitosan0.5 showed the highest numerical average daily gain at 1.10 ± 0.25 g/day, followed by HwChitosan1.0 at 0.94 ± 0.13 g/day, LwChitosan0.25 at 0.91 ± 0.22 g/day, Control at 0.90 ± 0.06 g/day, and LwChitosan1.0 at 0.86 ± 0.16 g/day.

Specific growth rate ranged from 2.61 ± 0.73 to 2.96 ± 0.70%/day and was not significantly affected by dietary treatment (*p* = 0.914). The highest numerical value was observed in the LwChitosan0.5 group, followed by the HwChitosan1.0, Control, LwChitosan0.25, and LwChitosan1.0 groups, with values of 2.89 ± 0.39, 2.86 ± 0.59, 2.65 ± 0.20, and 2.61 ± 0.73%/day, respectively.

Length gain did not differ significantly among treatments (*p* = 0.401). Fish in the LwChitosan0.5 group showed the highest numerical length gain at 4.42 ± 0.71 cm/fish, followed by the HwChitosan1.0, Control, LwChitosan0.25, and LwChitosan1.0 groups, with values of 4.05 ± 0.33, 3.99 ± 0.64, 3.70 ± 0.30, and 3.63 ± 0.48 cm/fish, respectively.

Feed conversion ratio ranged from 0.86 ± 0.12 to 1.02 ± 0.18 and did not differ significantly among treatments (*p* = 0.588). The lowest numerical feed conversion ratio was observed in the LwChitosan0.5 group at 0.86 ± 0.12, followed by the LwChitosan0.25, HwChitosan1.0, Control, and LwChitosan1.0 groups, with values of 0.96 ± 0.03, 0.97 ± 0.04, 0.97 ± 0.14, and 1.02 ± 0.18, respectively. Although the LwChitosan0.5 group showed numerically higher growth performance and a lower feed conversion ratio, these differences were not statistically significant.

### 2.2. Serum Oxidative Stress Markers and Antioxidant Enzyme Activities

**Figure 2:**
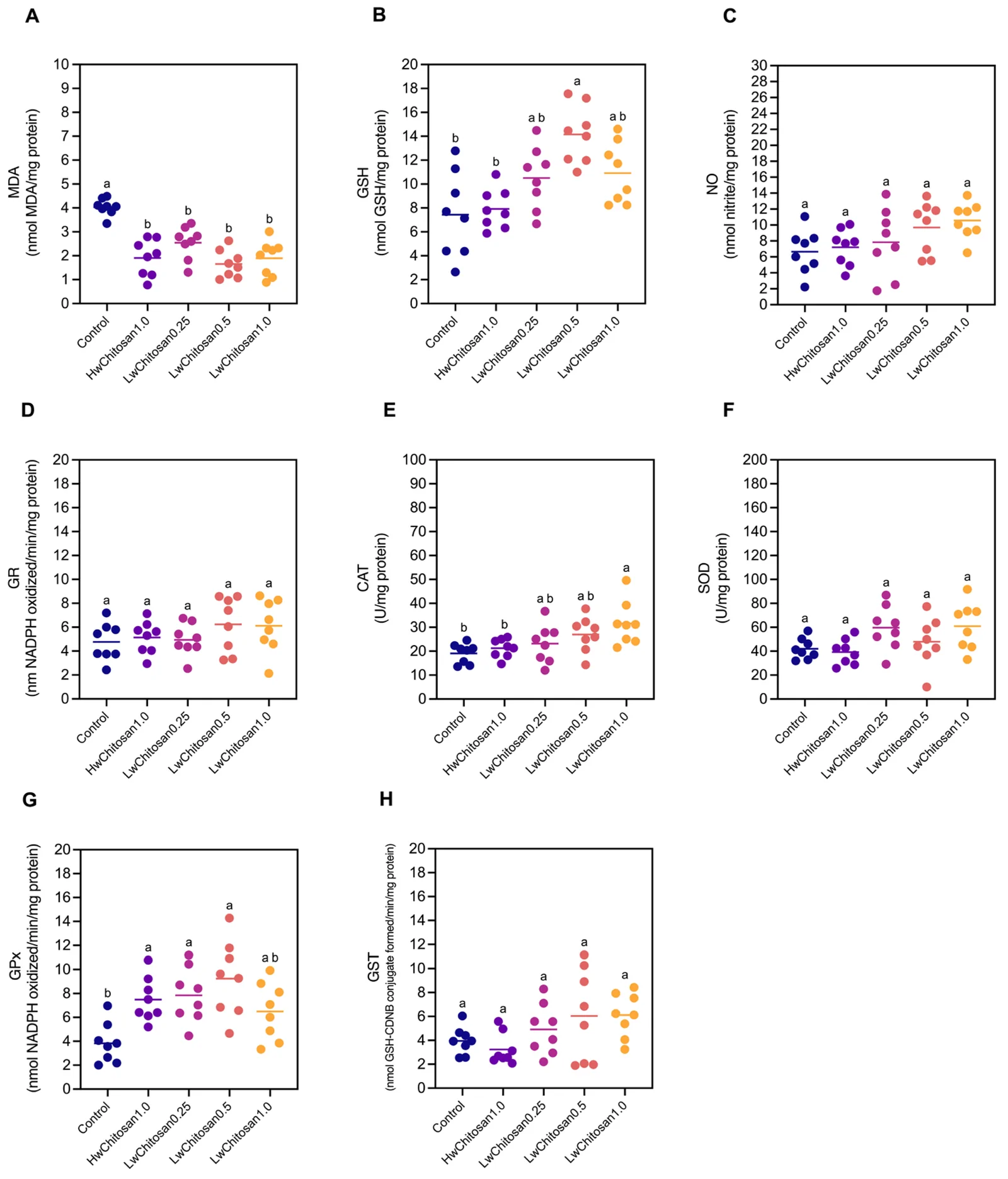
Serum oxidative stress markers and antioxidant enzyme activities of Asian seabass (*Lates calcarifer*) fed diets supplemented with high-molecular-weight chitosan or degraded low-molecular-weight chitosan for 4 weeks. (**A**) Malondialdehyde (MDA), (**B**) reduced glutathione (GSH), (**C**) nitric oxide (NO), (**D**) glutathione reductase (GR), (**E**) catalase (CAT), (**F**) superoxide dismutase (SOD), (**G**) glutathione peroxidase (GPx), and (**H**) glutathione-S-transferase (GST). Values are presented as mean ± SD (*n* = 8 fish per treatment; two fish from each of four replicate tanks). Individual fish were analyzed as biological samples, with tank included as a random effect. Different lowercase letters above bars indicate significant differences among treatments based on one-way ANOVA followed by Tukey’s HSD test (*p* < 0.05).

The effects of dietary chitosan supplementation on serum oxidative stress markers and antioxidant enzyme activities are presented in Figure 2A–H. Dietary treatment significantly affected serum MDA, GSH, CAT, GPx, and SOD levels, whereas no significant overall effects were detected for NO, GR, or GST.

Serum MDA levels differed significantly among treatments (*p* < 0.0001). The Control group showed the highest MDA level at 4.04 ± 0.35 nmol MDA/mg protein. Significantly lower values were observed in fish fed HwChitosan1.0, LwChitosan0.25, LwChitosan0.5, and LwChitosan1.0, with respective values of 1.91 ± 0.76, 2.55 ± 0.69, 1.66 ± 0.57, and 1.90 ± 0.73 nmol MDA/mg protein. No significant differences were detected among the chitosan-supplemented groups.

Serum GSH levels were also significantly affected by dietary treatment (*p* < 0.0001). Fish fed LwChitosan0.5 showed the highest GSH level at 14.15 ± 2.40 nmol GSH/mg protein, which was significantly higher than the levels recorded in the Control and HwChitosan1.0 groups at 7.45 ± 3.56 and 7.92 ± 1.66 nmol GSH/mg protein, respectively. The LwChitosan0.25 and LwChitosan1.0 groups showed intermediate values of 10.51 ± 2.59 and 10.92 ± 2.54 nmol GSH/mg protein, respectively, and did not differ significantly from either the LwChitosan0.5 group or the Control and HwChitosan1.0 groups.

Serum CAT activity differed significantly among treatments (*p* = 0.0062). The highest CAT activity was observed in the LwChitosan1.0 group at 31.64 ± 9.12 U/mg protein, which was significantly higher than the activities recorded in the Control and HwChitosan1.0 groups at 19.08 ± 4.11 and 21.22 ± 3.88 U/mg protein, respectively. The LwChitosan0.25 and LwChitosan0.5 groups showed intermediate activities of 23.13 ± 7.94 and 27.01 ± 7.26 U/mg protein, respectively, with no significant differences from the other groups.

Serum GPx activity was significantly affected by dietary treatment (*p* = 0.0008). The Control group showed the lowest GPx activity at 3.83 ± 1.68 nmol NADPH oxidized/min/mg protein. Significantly higher activities were observed in the HwChitosan1.0, LwChitosan0.25, and LwChitosan0.5 groups, with respective values of 7.49 ± 1.85, 7.85 ± 2.28, and 9.25 ± 3.13 nmol NADPH oxidized/min/mg protein. The LwChitosan1.0 group showed an intermediate activity of 6.50 ± 2.39 nmol NADPH oxidized/min/mg protein and did not differ significantly from either the Control group or the other chitosan-supplemented groups.

Serum SOD activity showed a significant overall treatment effect (*p* = 0.0308). Mean SOD activity was highest in the LwChitosan1.0 group at 60.96 ± 19.62 U/mg protein, followed by LwChitosan0.25 at 59.63 ± 18.43 U/mg protein, LwChitosan0.5 at 47.91 ± 20.08 U/mg protein, Control at 41.97 ± 8.68 U/mg protein, and HwChitosan1.0 at 39.28 ± 10.56 U/mg protein.

No significant differences were detected among treatments for serum NO levels (*p* = 0.0664). Mean nitrite levels increased numerically from 6.64 ± 2.75 nmol nitrite/mg protein in the Control group to 10.56 ± 2.24 nmol nitrite/mg protein in the LwChitosan1.0 group. The HwChitosan1.0, LwChitosan0.25, and LwChitosan0.5 groups showed values of 7.20 ± 2.29, 7.84 ± 4.23, and 9.70 ± 3.22 nmol nitrite/mg protein, respectively.

Similarly, serum GR activity was not significantly affected by dietary treatment (*p* = 0.348). Mean GR activity ranged from 4.77 ± 1.58 nmol NADPH oxidized/min/mg protein in the Control group to 6.24 ± 2.29 nmol NADPH oxidized/min/mg protein in the LwChitosan0.5 group. The HwChitosan1.0, LwChitosan0.25, and LwChitosan1.0 groups showed respective activities of 5.14 ± 1.35, 4.94 ± 1.35, and 6.12 ± 2.21 nmol NADPH oxidized/min/mg protein.

Serum GST activity also did not differ significantly among treatments (*p* = 0.0578). The lowest numerical GST activity was observed in the HwChitosan1.0 group at 3.23 ± 1.30 nmol GSH-CDNB conjugate formed/min/mg protein, whereas the highest activity was found in the LwChitosan1.0 group at 6.12 ± 1.84 nmol GSH-CDNB conjugate formed/min/mg protein. The Control, LwChitosan0.25, and LwChitosan0.5 groups showed respective values of 3.96 ± 1.14, 4.91 ± 2.10, and 6.04 ± 3.83 nmol GSH-CDNB conjugate formed/min/mg protein.

### 2.3. Humoral Immune Responses

**Figure 3:**
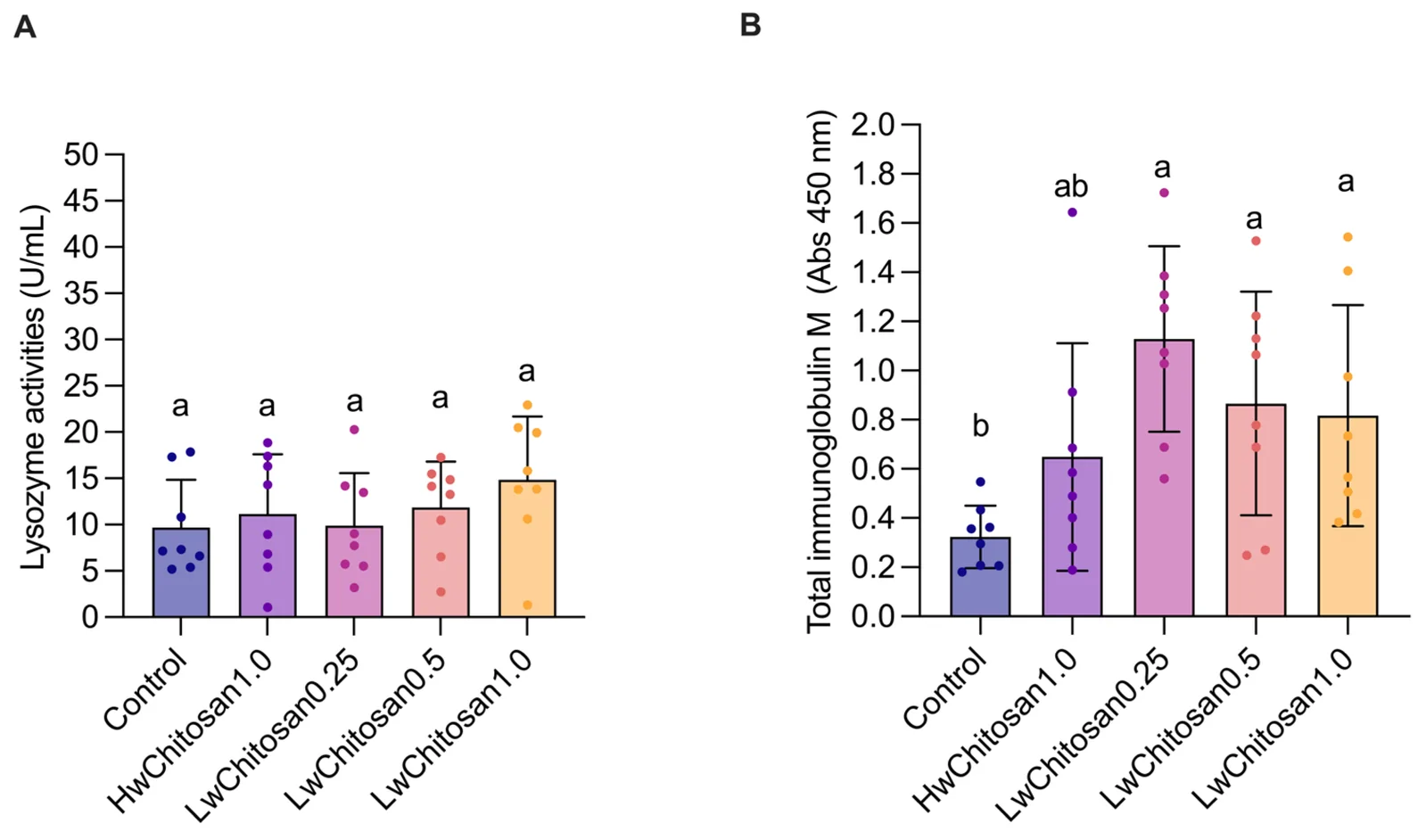
Humoral immune responses of Asian seabass (*Lates calcarifer*) fed diets supplemented with high-molecular-weight chitosan or degraded low-molecular-weight chitosan for 4 weeks. (**A**) Serum lysozyme activity and (**B**) total serum immunoglobulin M (IgM) level. Values are presented as mean ± SD (*n* = 8 fish per treatment; two fish from each of four replicate tanks). Individual fish were analyzed as biological samples, with tank included as a random effect. Different lowercase letters above bars indicate significant differences among treatments (*p* < 0.05).

The effects of dietary chitosan supplementation on serum lysozyme activity and total serum immunoglobulin M levels are presented in Figure 3A,B. Dietary treatment did not significantly affect serum lysozyme activity, whereas total serum IgM levels differed significantly among treatments.

Serum lysozyme activity showed no significant differences among dietary treatments (*p* = 0.421). The highest numerical activity was observed in the LwChitosan1.0 group at 14.84 ± 6.83 U/mL, followed by LwChitosan0.5 at 11.84 ± 4.96 U/mL, HwChitosan1.0 at 11.14 ± 6.48 U/mL, LwChitosan0.25 at 9.89 ± 5.68 U/mL, and Control at 9.71 ± 5.17 U/mL (Figure 3A).

In contrast, total serum IgM levels were significantly affected by dietary treatment (*p* = 0.0048). The Control group showed the lowest IgM level at 0.32 ± 0.13 absorbance units at 450 nm, whereas the highest level was observed in the LwChitosan0.25 group at 1.13 ± 0.38 absorbance units. Total serum IgM in the LwChitosan0.25 group was significantly higher than that in the Control group, with an adjusted *p* value of 0.0022. The HwChitosan1.0, LwChitosan0.5, and LwChitosan1.0 groups showed intermediate IgM levels of 0.65 ± 0.46, 0.87 ± 0.46, and 0.82 ± 0.45 absorbance units, respectively. These intermediate groups did not differ significantly from either the Control or LwChitosan0.25 groups (Figure 3B).

### 2.4. Gene Expression Profiles

#### 2.4.1. Liver Gene Expression Profiles

**Figure 4:**
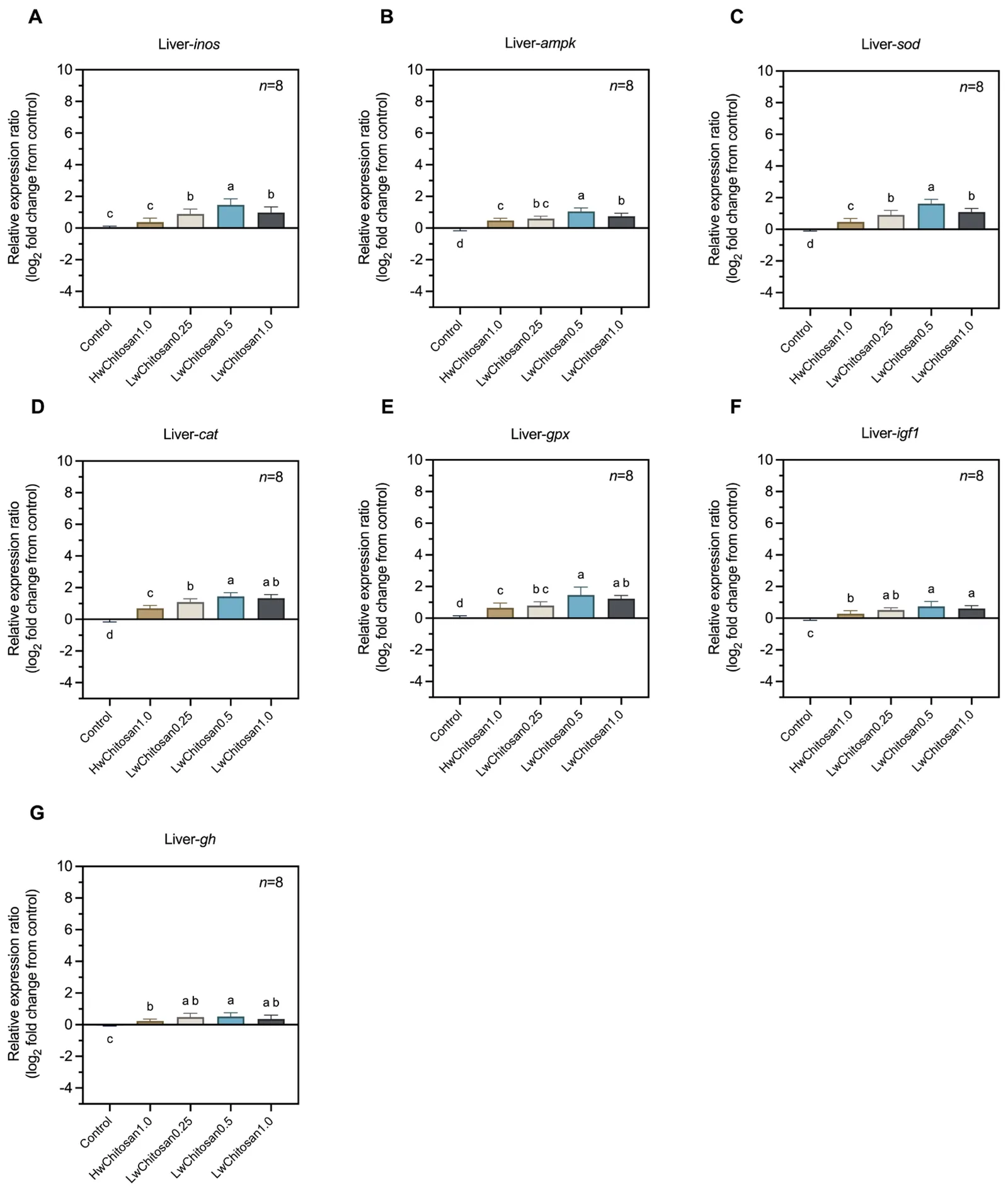
Relative hepatic gene expression profiles of Asian seabass (*Lates calcarifer*) fed diets supplemented with high-molecular-weight chitosan or degraded low-molecular-weight chitosan for 4 weeks. Relative expression levels of liver genes associated with inflammatory response, metabolic regulation, antioxidant defense, and growth signaling: (**A**) *inos*, (**B**) *ampk*, (**C**) *sod*, (**D**) *cat*, (**E**) *gpx*, (**F**) *igf1*, and (**G**) *gh*. Gene expression values are presented as log_2_ fold change from the control group. Values are presented as mean ± SD (*n* = 8 fish per treatment; two fish from each of four replicate tanks). Individual fish were analyzed as biological samples, with tank included as a random effect. Different lowercase letters above bars indicate significant differences among treatments based on one-way ANOVA followed by Tukey’s HSD test (*p* < 0.05).

The relative expression levels of liver genes involved in inflammatory regulation, energy metabolism, antioxidant defense, and growth regulation are shown in Figure 4A–G. Dietary supplementation with chitosan significantly modulated the hepatic expression of all analyzed genes, including *inos*, *ampk*, *sod*, *cat*, *gpx*, *igf1*, and *gh.*

For genes associated with inflammatory and metabolic regulation, *inos* and *ampk* expression were significantly upregulated in chitosan-supplemented groups compared with the control group. The highest expression was consistently observed in fish fed LwChitosan0.5, followed by LwChitosan1.0 and LwChitosan0.25, indicating a stronger hepatic response to degraded low-molecular-weight chitosan than to high-molecular-weight chitosan. For *ampk*, the LwChitosan0.5 group showed the highest expression level, while all chitosan-treated groups were significantly higher than the control group.

The antioxidant-related genes *sod*, *cat*, and *gpx* were also significantly enhanced by dietary chitosan supplementation. Among these genes, the strongest upregulation was generally detected in the LwChitosan0.5 group. For *sod,* expression was highest in LwChitosan0.5, followed by LwChitosan1.0 and LwChitosan0.25, with all supplemented groups showing significantly higher expression than the control. Similarly, *cat* and *gpx* expression were markedly increased in fish fed degraded low-molecular-weight chitosan, especially at 0.5%.

Growth-related genes showed a similar positive response to chitosan supplementation. Hepatic *igf1* expression was significantly higher in all chitosan-supplemented groups than in the control group, with the highest numerical value observed in LwChitosan0.5. Expression of *gh* was also significantly increased in all supplemented groups compared with the control, with LwChitosan0.5 and LwChitosan0.25 showing the strongest responses.

#### 2.4.2. Head Kidney Immune-Related Gene Expression Profiles

**Figure 5:**
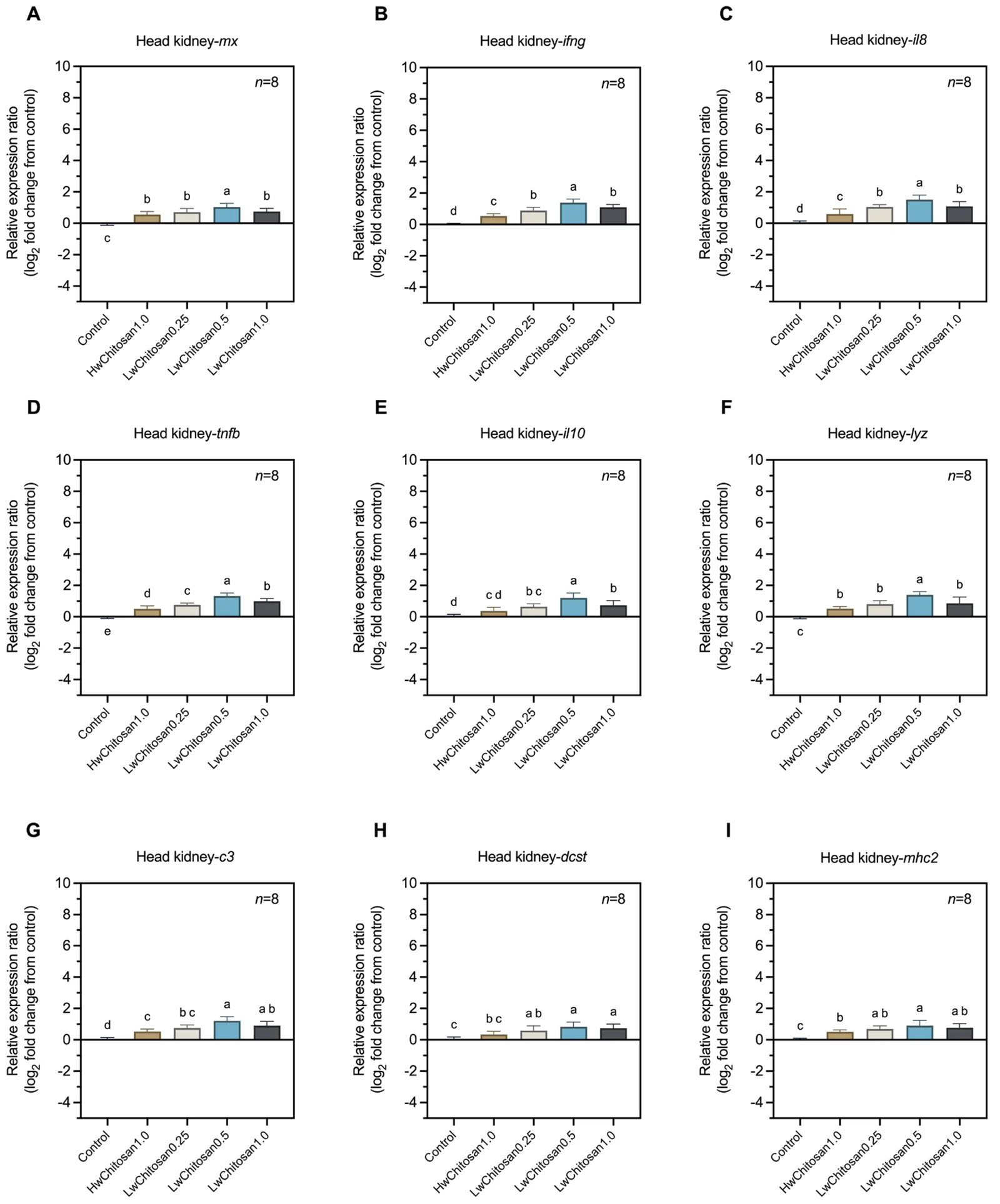
Relative expression profiles of immune-related genes in the head kidney of Asian seabass (*Lates calcarifer*) fed diets supplemented with high-molecular-weight chitosan or degraded low-molecular-weight chitosan for 4 weeks. Relative expression levels of genes associated with antiviral response, inflammatory regulation, innate immunity, complement activation, dendritic cell-associated signaling, and antigen presentation: (**A**) *mx*, (**B**) *ifng*, (**C**) *il8*, (**D**) *tnfb*, (**E**) *il10*, (**F**) *lyz*, (**G**) *c3*, (**H**) *dcst*, and (**I**) *mhc2*. Gene expression values are presented as log_2_ fold change from the control group. Values are presented as mean ± SD (*n* = 8 fish per treatment; two fish from each of four replicate tanks). Individual fish were analyzed as biological samples, with tank included as a random effect. Different lowercase letters above bars indicate significant differences among treatments based on one-way ANOVA followed by Tukey’s HSD test (*p* < 0.05).

The relative expression profiles of immune-related genes in the head kidney are shown in Figure 5A–I. Dietary supplementation with chitosan significantly modulated the expression of all analyzed genes, including *mx*, *ifng*, *il8*, *tnfb*, *il10*, *lyz*, *c3*, *dcst*, and *mhc2*.

Expression of antiviral and inflammatory response genes, including *mx*, *ifng*, *il8*, and *tnfb*, was significantly upregulated in chitosan-supplemented groups compared with the control group. Among treatments, LwChitosan0.5 consistently induced the strongest expression response, followed by LwChitosan1.0 and LwChitosan0.25. For *mx*, *ifng*, and *il8*, the LwChitosan0.5 group showed the highest expression level, whereas the control group showed the lowest expression. *tnfb* expression showed a clear dose-responsive pattern, with the highest expression in LwChitosan0.5, followed by LwChitosan1.0, LwChitosan0.25, HwChitosan1.0, and control.

The anti-inflammatory and innate immune-related genes *il10* and *lyz* were also significantly enhanced by chitosan supplementation. The highest expression of both genes was detected in fish fed LwChitosan0.5.

Complement and antigen-presentation-related genes showed similar responses. Expression of *c3*, *dcst*, and *mhc2* was significantly higher in chitosan-fed fish than in the control group, with the strongest upregulation generally observed in the LwChitosan0.5 group.

#### 2.4.3. Spleen Immune-Related Gene Expression Profiles

**Figure 6:**
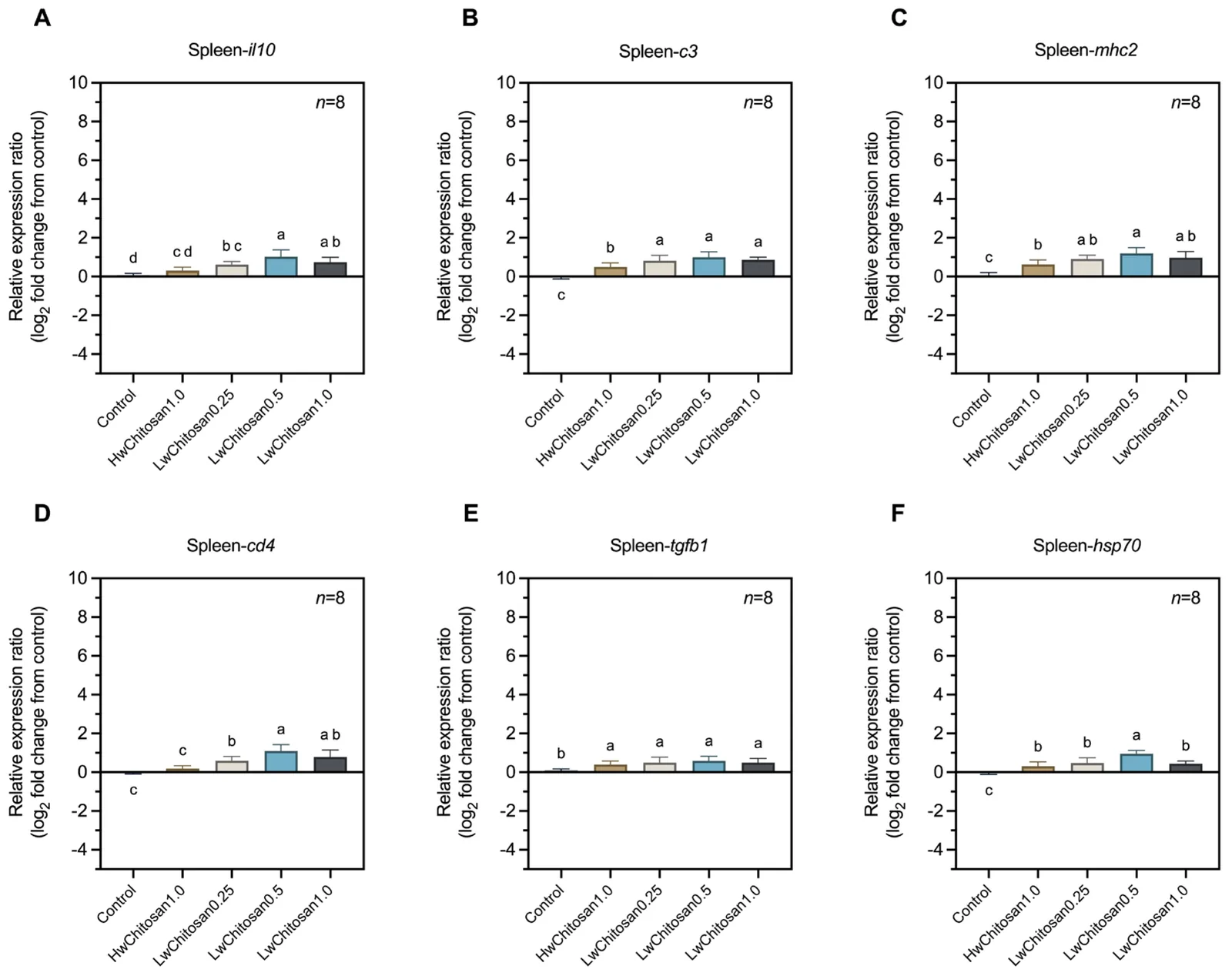
Relative expression profiles of immune- and stress-related genes in the spleen of Asian seabass (*Lates calcarifer*) fed diets supplemented with high-molecular-weight chitosan or degraded low-molecular-weight chitosan for 4 weeks. Relative expression levels of spleen genes associated with immune regulation, complement activation, antigen presentation, T-cell response, anti-inflammatory signaling, and stress response: (**A***) il10*, (**B**) *c3*, (**C**) *mhc2*, (**D**) *cd4*, (**E**) *tgfb1*, and (**F**) *hsp70*. Gene expression values are presented as log_2_ fold change from the control group. Values are presented as mean ± SD (*n* = 8 fish per treatment; two fish from each of four replicate tanks). Individual fish were analyzed as biological samples, with tank included as a random effect. Different lowercase letters above bars indicate significant differences among treatments based on one-way ANOVA followed by Tukey’s HSD test (*p* < 0.05).

The relative expression levels of immune- and stress-related genes in the spleen are shown in Figure 6A–F. Dietary chitosan supplementation significantly modulated the expression of all analyzed genes, including *il10*, *c3*, *mhc2*, *cd4*, *tgfb1*, and *hsp70*.

Expression of *il10*, an immune-regulatory cytokine, was significantly increased in fish fed low-molecular-weight chitosan compared with the control group. The highest expression was observed in the LwChitosan0.5 group, followed by LwChitosan1.0 and LwChitosan0.25, whereas the control group showed the lowest expression level.

The complement-related gene *c3* was significantly upregulated in all chitosan-supplemented groups compared with the control group. The LwChitosan0.25, LwChitosan0.5, and LwChitosan1.0 groups showed significantly higher expression than HwChitosan1.0.

Genes associated with adaptive immunity and antigen presentation also responded positively to chitosan supplementation. Expression of *mhc2* was significantly increased in all chitosan-supplemented groups, with the highest numerical expression observed in LwChitosan0.5. Similarly, *cd4* expression was highest in the LwChitosan0.5 group, followed by LwChitosan1.0 and LwChitosan0.25, while the control and HwChitosan1.0 groups showed lower expression.

Expression of *tgfb1* was significantly higher in all chitosan-supplemented groups than in the control group; however, no significant differences were observed among the chitosan-treated groups. In contrast, *hsp70* showed a stronger dose-related pattern, with the highest expression detected in the LwChitosan0.5 group, followed by the other chitosan-supplemented groups.

#### 2.4.4. Gills Gene Expression Profiles

**Figure 7:**
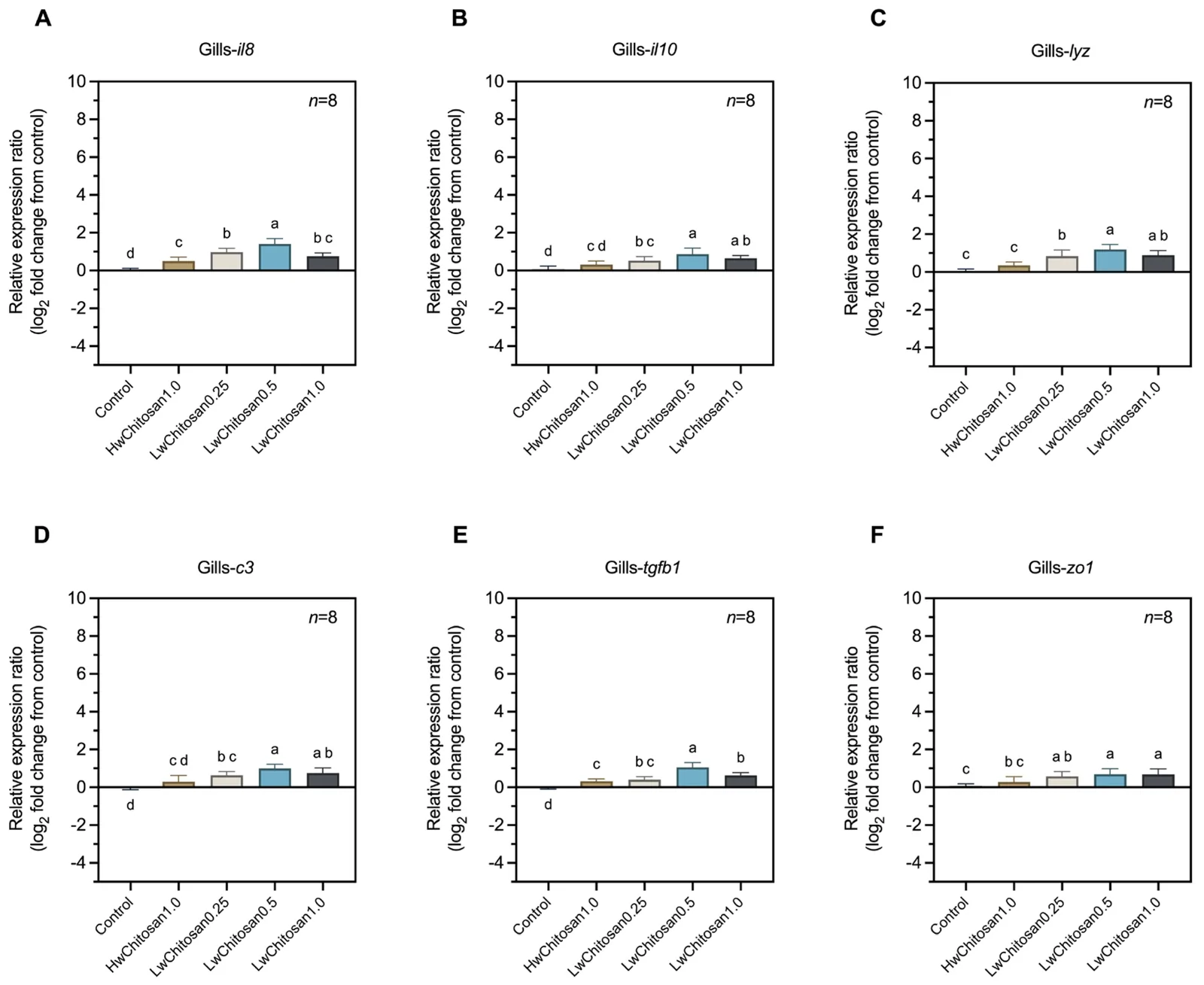
Relative expression profiles of immune- and epithelial barrier-related genes in the gills of Asian seabass (*Lates calcarifer*) fed diets supplemented with high-molecular-weight chitosan or degraded low-molecular-weight chitosan for 4 weeks. Relative expression levels of gill genes associated with inflammatory response, immune regulation, innate immunity, complement activation, anti-inflammatory signaling, and epithelial barrier integrity: (**A**) *il8*, (**B**) *il10*, (**C**) *lyz*, (**D**) *c3*, (**E**) *tgfb1*, and (**F**) *zo1*. Gene expression values are presented as log_2_ fold change from the control group. Values are presented as mean ± SD (*n* = 8 fish per treatment; two fish from each of four replicate tanks). Individual fish were analyzed as biological samples, with tank included as a random effect. Different lowercase letters above bars indicate significant differences among treatments based on one-way ANOVA followed by Tukey’s HSD test (*p* < 0.05).

The relative expression profiles of immune- and epithelial barrier-related genes in the gills are shown in Figure 7A–F. Dietary chitosan supplementation significantly modulated the expression of all analyzed genes, including *il8*, *il10*, *lyz*, *c3*, *tgfb1*, and *zo1*.

Expression of the inflammatory cytokine *il8* was significantly increased in fish fed degraded low-molecular-weight chitosan compared with the control group. The highest expression was observed in the LwChitosan0.5 group, followed by LwChitosan1.0 and LwChitosan0.25. The HwChitosan1.0 group showed a moderate increase, but the response was lower than that of the LwChitosan0.5 group.

The immune-regulatory cytokines *il10* and *tgfb1* were also significantly upregulated by dietary chitosan supplementation. For both genes, the LwChitosan0.5 group showed the highest expression level, while the control group showed the lowest expression.

Innate immune genes *lyz* and *c3* were significantly enhanced in fish fed low-molecular-weight chitosan. The highest expression of both genes was recorded in the LwChitosan0.5 group, followed by LwChitosan1.0 and LwChitosan0.25.

Expression of the tight-junction-related gene *zo1* was significantly higher in fish fed degraded low-molecular-weight chitosan than in the control group. The LwChitosan0.5 and LwChitosan1.0 groups showed the highest expression values.

#### 2.4.5. Intestinal Immune- and Barrier-Related Gene Expression Profiles

**Figure 8:**
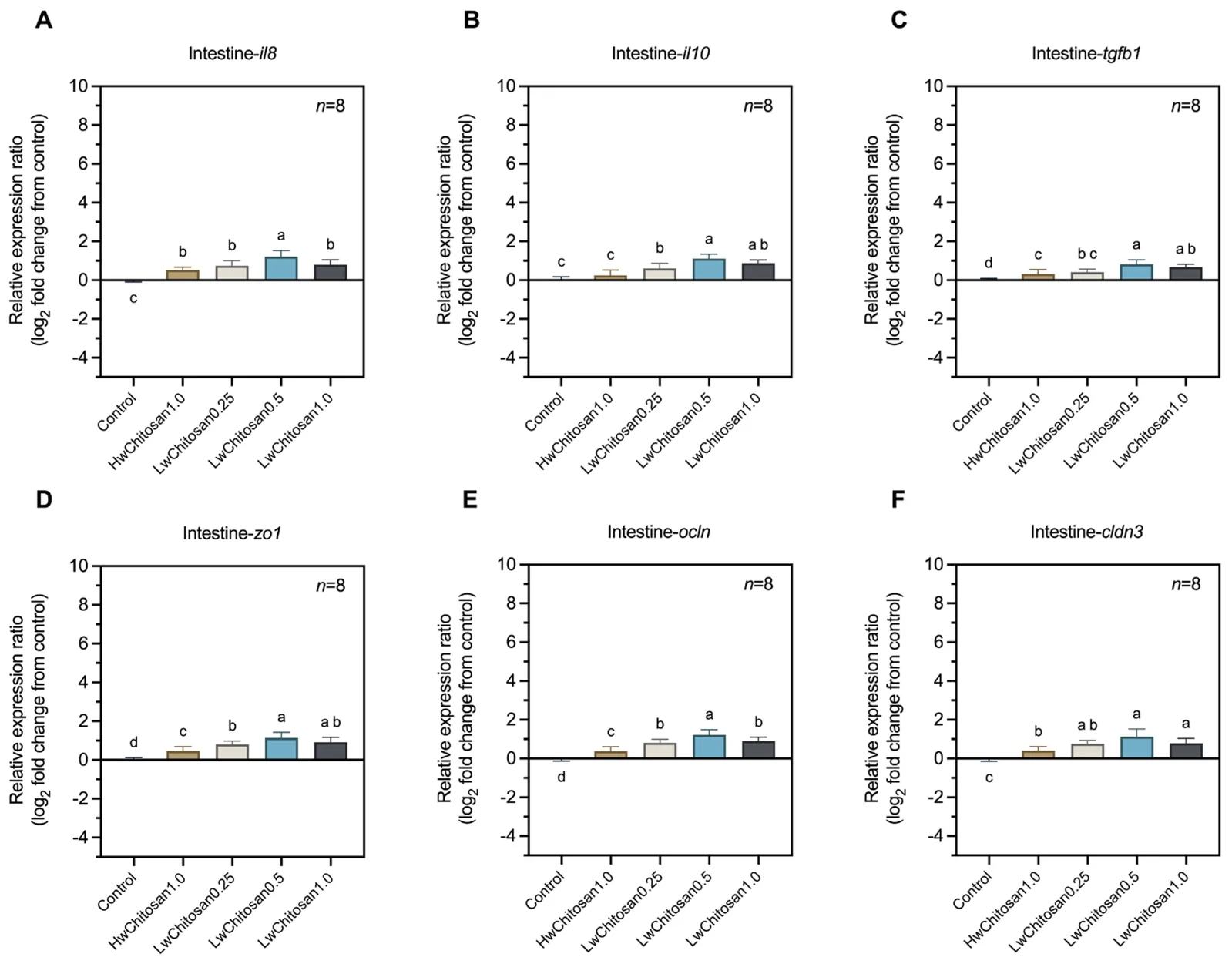
Relative expression profiles of immune- and epithelial barrier-related genes in the intestine of Asian seabass (*Lates calcarifer*) fed diets supplemented with high-molecular-weight chitosan or degraded low-molecular-weight chitosan for 4 weeks. Relative expression levels of intestinal genes associated with inflammatory response, immune regulation, anti-inflammatory signaling, and tight-junction integrity: (**A**) *il8*, (**B**) *il10*, (**C**) *tgfb1*, (**D**) *zo1*, (**E**) *ocln*, and (**F**) *cldn3*. Gene expression values are presented as log_2_ fold change from the control group. Values are presented as mean ± SD (*n* = 8 fish per treatment; two fish from each of four replicate tanks). Individual fish were analyzed as biological samples, with tank included as a random effect. Different lowercase letters above bars indicate significant differences among treatments based on one-way ANOVA followed by Tukey’s HSD test (*p* < 0.05).

The relative expression profiles of immune- and epithelial barrier-related genes in the intestine are shown in Figure 8A–F. Dietary chitosan supplementation significantly modulated the expression of all analyzed intestinal genes, including *il8*, *il10*, *tgfb1*, *zo1*, *ocln*, and *cldn3*.

Expression of the inflammatory cytokine *il8* was significantly increased in all chitosan-supplemented groups compared with the control group. The highest expression was observed in the LwChitosan0.5 group, followed by LwChitosan1.0, LwChitosan0.25, and HwChitosan1.0.

The immune-regulatory cytokines *il10* and *tgfb1* were also significantly upregulated following dietary chitosan supplementation. For both genes, expression was highest in the LwChitosan0.5 group and lowest in the control group.

Genes associated with epithelial barrier integrity, including *zo1*, *ocln*, and *cldn3*, were significantly increased in fish fed chitosan-supplemented diets. The strongest upregulation of *zo1* and *cldn3* was observed in the LwChitosan0.5 group, while *ocln* expression was also highest in this group and significantly higher than the other treatments.

### 2.5. Survival After Photobacterium damselae Challenge

**Figure 9:**
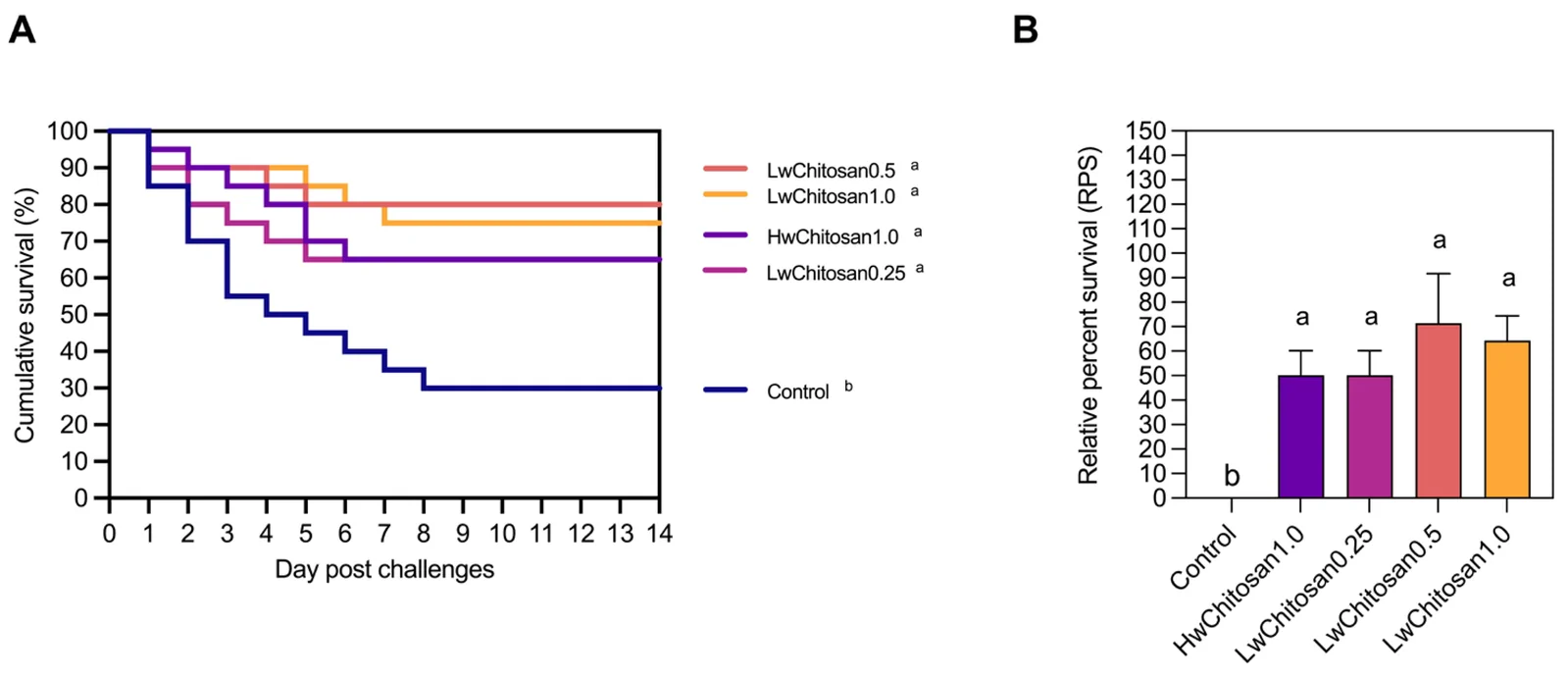
Cumulative survival and relative percent survival of Asian seabass (*Lates calcarifer*) fed chitosan-supplemented diets following *Photobacterium damselae* challenge. Each treatment included four replicate tanks with 10 fish per tank, for a total of 40 fish per treatment. (**A**) Kaplan–Meier cumulative survival curves over 14 days post-challenge and (**B**) relative percent survival (RPS). Different lowercase letters above bars indicate significant differences among treatments based on one-way ANOVA followed by Tukey’s HSD test (*p* < 0.05).

The cumulative survival and relative percent survival (RPS) of Asian seabass after *Photobacterium damselae* challenge are shown in Figure 9A,B. The control group showed the lowest cumulative survival, declining rapidly during the first week post-challenge and reaching approximately 30% survival by the end of the 14-day observation period. In contrast, fish fed chitosan-supplemented diets showed improved survival, particularly in the LwChitosan0.5 and LwChitosan1.0 groups.

Kaplan–Meier survival analysis showed that fish fed HwChitosan1.0, LwChitosan0.5, and LwChitosan1.0 had significantly higher survival than the control group. The survival curve of HwChitosan1.0 differed significantly from the control group based on the log-rank test (*p* = 0.0250), while stronger differences were observed for LwChitosan0.5 (*p* = 0.0023) and LwChitosan1.0 (*p* = 0.0038). The LwChitosan0.25 group showed improved survival compared with the control, but this difference was not statistically significant (*p* = 0.0532).

The median survival time of the control group was 4.5 days, whereas median survival was undefined in all chitosan-supplemented groups because survival remained above 50% throughout the challenge period (Figure 9A).

RPS analysis confirmed that dietary chitosan supplementation enhanced protection against *Photobacterium damselae*. Fish fed HwChitosan1.0 and LwChitosan0.25 showed 50.00% RPS, while higher values were recorded in the LwChitosan0.5 and LwChitosan1.0 groups at 71.43% and 64.29%, respectively. All chitosan-supplemented groups showed significantly higher RPS than the Control group, with the highest numerical protection observed in fish fed LwChitosan0.5 (Figure 9B).

No significant differences were detected among the chitosan-supplemented groups for survival curve comparisons, indicating that both high-molecular-weight chitosan and degraded low-molecular-weight chitosan improved disease resistance compared with the unsupplemented control. However, the strongest protective response was consistently observed in fish fed 0.5% degraded low-molecular-weight chitosan.

## 3. Discussion

The biological effects of chitosan are influenced by molecular weight, degree of deacetylation, solubility, viscosity, charge density, and the accessibility of protonated amino groups. Reduction in polymer-chain length generally decreases solution viscosity and improves dispersion, which may increase the accessibility of chitosan at gastrointestinal and mucosal interfaces. Greater exposure of positively charged amino groups may facilitate interactions with negatively charged microbial and epithelial surfaces, while smaller chitosan fragments may interact more readily with immune cells and cellular processes involved in inflammatory and redox regulation. In parallel, the antioxidant effects of chitosan may involve direct radical-scavenging and metal-chelating properties together with modulation of endogenous antioxidant defenses. Recent studies in aquatic species have similarly demonstrated that dietary chitosan, chitosan nanoparticles, and chitosan oligosaccharides can influence antioxidant status, immune responses, intestinal health, and resistance to bacterial challenge. These characteristics provide a plausible explanation for the broader responses observed with the approximately 10 kDa degraded preparation compared with the approximately 85 kDa native preparation in the present study. Nevertheless, gastrointestinal solubility, receptor interactions, cellular uptake, radical-scavenging activity, immune-cell populations, and protein abundance were not directly measured. Therefore, the present findings demonstrate an association between molecular-weight reduction and enhanced biological responsiveness but do not establish a specific causal molecular mechanism [13,14,16].

Reduction in polymer-chain length generally decreases solution viscosity and may improve dispersion in the gastrointestinal environment. This can increase contact between the cationic polymer and negatively charged microbial or mucosal surfaces. Low-molecular-weight chitosan and chitosan oligosaccharides may also interact with immune cells or pattern-recognition processes and can influence redox-sensitive cellular responses [17,20]. In parallel, antioxidant effects may arise from radical-scavenging or metal-chelating properties and from changes in endogenous antioxidant responses. These mechanisms could collectively explain why the degraded preparation produced broader responses at a lower inclusion level than native chitosan. Nevertheless, these mechanisms remain hypotheses in the context of the present experiment. Solubility in the gastrointestinal tract, radical-scavenging activity, receptor binding, immune-cell composition, protein abundance, and epithelial permeability were not directly measured. The present results therefore support associations among molecular-weight reduction, serum antioxidant and immune measurements, transcriptional responses, and post-challenge survival, but they do not establish a single causal molecular pathway.

In addition, the present study demonstrated that dietary supplementation with synergistically degraded low-molecular-weight chitosan improved redox balance, humoral immunity, tissue-specific immune gene expression, mucosal barrier-related responses, and resistance of Asian seabass (*Lates calcarifer*) to *P. damselae*. The degraded low-molecular-weight chitosan used in this study was produced by γ-irradiation at 10 kGy in combination with 0.25% H_2_O_2_, reducing the molecular weight to approximately 10 kDa, whereas the native non-degraded chitosan had an approximate molecular weight of 85 kDa. This preparation strategy is consistent with previous reports showing that γ-irradiation combined with hydrogen peroxide is an efficient method for chitosan depolymerization because both treatments act synergistically to cleave polymer chains while largely preserving key functional groups. Hien et al. reported that γ-irradiation in the presence of H_2_O_2_ increased the degradation efficiency of chitosan and was suitable for preparing oligochitosan [25], while Kang et al. also demonstrated a synergistic degradation effect between γ-radiation and hydrogen peroxide [26].

Dietary chitosan did not significantly affect final body weight, weight gain, average daily gain, specific growth rate, length gain, or FCR during the four-week trial. Therefore, the numerically higher means observed in the LwChitosan0.5 group should not be interpreted as evidence of growth promotion. Improvements in antioxidant or immune measurements do not necessarily increase somatic growth because nutrients and metabolic resources can be allocated to cellular maintenance, redox regulation, and immune preparedness without producing a measurable change in biomass. In addition, the fish were maintained under stable water-quality conditions and received a nutritionally adequate commercial diet. Under these conditions, limited physiological stress and adequate nutrient availability may have reduced the opportunity for a functional additive to improve growth. The short experimental period and variability among replicate tanks could also have limited the ability to detect modest growth effects. These findings are consistent with the broader literature showing that the growth response to dietary chitosan is variable and depends on fish species, molecular characteristics, inclusion level, diet composition, culture conditions, and feeding duration [17,20]. A previous 60-day study in Asian seabass reported beneficial hematological, immune, and post-challenge responses at selected chitosan levels [27]. The shorter duration of the present study may explain why health-related changes and improved post-challenge survival were observed without a statistically significant growth response. Accordingly, the present material should be considered a potential short-term health-supporting additive rather than a demonstrated growth promoter.

Previous work in Asian seabass reported stronger growth- and immunity-related responses after a longer feeding period. Ranjan et al. evaluated dietary chitosan at 0, 5, 10, and 20 g/kg diet for 60 days in Asian seabass and found that chitosan-fed fish showed improved hematological and innate immune parameters, with 10 g/kg producing the strongest response and higher post-challenge survival against *Vibrio anguillarum* [27]. In comparison, the present study used degraded low-molecular-weight chitosan and a shorter 4-week feeding duration but still observed improved antioxidant status, immune gene expression, total serum IgM, and disease resistance. This indicates that molecular-weight reduction may improve chitosan solubility, reduce viscosity, and facilitate its interaction with biological interfaces, potentially allowing measurable immunonutritional effects during a shorter feeding period [13,16]. Despite the improvements observed in several antioxidant, immune, transcriptional, and post-challenge endpoints, dietary chitosan did not significantly affect final body weight, weight gain, average daily gain, specific growth rate, length gain, or FCR during the four-week feeding trial. Therefore, the numerically higher growth measurements observed in LwChitosan0.5 should not be interpreted as evidence of growth promotion. Improved antioxidant status or immune preparedness does not necessarily result in increased somatic growth because nutrients and metabolic resources may be allocated toward cellular maintenance, redox regulation, epithelial integrity, and immune function without producing a measurable increase in biomass. In addition, the fish were maintained under relatively stable environmental conditions and received a nutritionally adequate commercial diet, potentially limiting the scope for an additional functional ingredient to improve growth. The four-week duration may also have been insufficient for modest physiological improvements to accumulate into detectable differences in body growth or feed utilization. Thus, under the conditions evaluated here, the principal effects of low-molecular-weight chitosan appear to involve health support and disease resilience rather than direct growth enhancement.

The reduction in serum malondialdehyde observed in all chitosan-supplemented groups indicates that both native and degraded chitosan reduced lipid peroxidation. MDA is a widely used end-product marker of lipid peroxidation and is commonly measured to assess oxidative damage in fish and other aquatic organisms [28]; therefore, the lower MDA values in chitosan-fed fish suggest improved protection against oxidative stress. This finding agrees with studies in other aquatic animals showing that chitin, chitosan, and chitosan derivatives can reduce oxidative damage and regulate antioxidant defenses. Niu et al. reported that dietary chitin and chitosan reduced oxidative stress markers, including MDA, in shrimp exposed to low dissolved oxygen stress [23]. In the present study, reduced MDA was accompanied by increased GSH and GPx activity, especially in the LwChitosan0.5 group, suggesting that low-molecular-weight chitosan enhanced glutathione-dependent antioxidant defense.

The antioxidant response observed here is also consistent with recent fish studies using chitosan or chitosan nanoparticles. Hossam-Elden et al. reported that dietary chitosan nanoparticles improved antioxidant defenses, immunity, and disease resistance in Nile tilapia, with beneficial effects on health and bacterial resistance [19]. Similarly, Zhang et al. reported that dietary chitosan improved antioxidant-related parameters in fish, including CAT, GST, GSH-Px, and total antioxidant capacity, while reducing oxidative damage [29]. In the present study, the strongest antioxidant response was not always dose-dependent. The LwChitosan0.5 group showed the most consistent improvement in GSH and GPx, whereas CAT activity was highest in the LwChitosan1.0 group. This suggests that 0.5% low-molecular-weight chitosan may be sufficient to stimulate glutathione-based antioxidant protection, while higher inclusion may not necessarily provide additional benefit for all antioxidant markers.

Humoral immune responses showed that total serum IgM was significantly increased, particularly in fish fed LwChitosan0.25, whereas lysozyme activity was not significantly different among treatments. This pattern suggests that dietary low-molecular-weight chitosan may more strongly influence antibody-related humoral responses than serum lysozyme activity under the present experimental conditions. The result partly differs from Ranjan et al., who reported significant enhancement of serum lysozyme and other innate immune parameters in Asian seabass fed chitosan for 60 days [27]. The difference may be explained by feeding duration, dose, molecular weight, pathogen challenge model, fish size, and sampling time. In the present study, lysozyme activity showed a numerical increase, especially in the LwChitosan1.0 group, but the variation among individuals may have prevented statistical significance.

The increase in total serum IgM supports the immunostimulatory role of chitosan in fish. IgM is the predominant systemic immunoglobulin in most teleost fish and contributes to pathogen recognition, agglutination, complement activation, and systemic humoral defense [30,31]. The elevated IgM response in the present study is consistent with the general conclusion that chitosan and chitosan derivatives can enhance immune readiness in aquatic animals. Abdel-Ghany and Salem reviewed multiple studies and concluded that dietary chitosan can improve fish immune responses and disease resistance, although the optimal inclusion level varies across species and experimental systems [17]. The stronger IgM response at LwChitosan0.25 than at higher doses may indicate a non-linear immunomodulatory effect, where a lower level of soluble low-molecular-weight chitosan is sufficient to activate humoral immunity, while higher doses may shift the response toward tissue-level innate, mucosal, and antioxidant pathways.

The liver gene expression results further support the antioxidant and metabolic roles of low-molecular-weight chitosan. Dietary chitosan significantly upregulated hepatic sod, cat, and gpx, which correspond well with serum antioxidant changes. These genes encode major components of the enzymatic antioxidant system: SOD converts superoxide radicals into hydrogen peroxide, CAT decomposes hydrogen peroxide, and GPx reduces hydrogen peroxide and lipid hydroperoxides using glutathione as an electron donor [32]. The strongest overall expression was observed in the LwChitosan0.5 group, suggesting that this dose effectively primed hepatic antioxidant defense. The upregulation of *ampk* suggests modulation of cellular energy-sensing pathways, because AMPK is a conserved cellular energy sensor that coordinates metabolic responses to changes in nutrient and energy availability and contributes to physiological adaptation to energetic stress in fish [33]. In addition, the upregulation of *igf1* and *gh* indicates that low-molecular-weight chitosan may support growth-related endocrine signaling, even though growth performance differences were not statistically significant during the 4-week trial.

The higher transcript abundance of hepatic antioxidant genes is comparable with previous transcriptional findings in Nile tilapia fed chitosan nanoparticles, where dietary supplementation enhanced antioxidant defenses and health-related gene expression [19]. However, the present study extends those observations to Asian seabass and shows that low-molecular-weight chitosan produced by γ-irradiation and H_2_O_2_ depolymerization can stimulate antioxidant-related transcription without requiring nanoparticle formulation. This is practically important because degraded chitosan may be easier to standardize and incorporate into feed than some nanoparticle preparations, while still providing improved solubility and biological activity.

The head kidney is a major hematopoietic, lymphoid, and endocrine organ in teleost fish and contains important immune-cell populations involved in innate and adaptive responses [34,35], and the present study showed broad upregulation of immune-related genes in this tissue, including *mx*, *ifng*, *il8*, *tnfb*, *il10*, *lyz*, *c3*, *dcst*, and *mhc2*. These genes represent antiviral signaling, inflammatory cytokine responses, innate antibacterial defense, complement activation, dendritic cell-associated activity, and antigen presentation. The transcriptional pattern in the LwChitosan0.5 group is consistent with increased immune readiness, although it does not by itself confirm functional activation of the corresponding systemic immune pathways. Previous studies have proposed that chitosan can act as an immunostimulant through interactions with immune cells and pattern-recognition pathways. In blunt snout bream, chitosan oligosaccharides induced immunomodulatory responses in head kidney macrophages [24]. The present data are consistent with this mechanism, as head kidney genes related to both inflammatory and antigen-presentation pathways were enhanced by dietary low-molecular-weight chitosan.

The teleost spleen is an important secondary lymphoid organ involved in blood filtration, antigen presentation, lymphocyte activation, and the initiation of adaptive immune responses [36]. The spleen gene expression profile also indicated improved systemic immune regulation. Genes associated with immune regulation (*il10*, *tgfb1*), complement (*c3*), antigen presentation (*mhc2*), T-cell response (*cd4*), and stress response (*hsp70*) were upregulated, particularly in the LwChitosan0.5 group. This suggests that low-molecular-weight chitosan did not simply induce inflammation but promoted a coordinated immune-ready state involving regulatory and adaptive immune markers. Similar immunomodulatory effects have been reported in fish and shrimp fed chitosan derivatives, where increased immune gene expression was associated with higher survival following bacterial challenge. Hossam-Elden et al. found that chitosan nanoparticles improved immunity and resistance to *Aeromonas veronii* in Nile tilapia [19].

The gills and intestine are major mucosal interfaces continuously exposed to waterborne microorganisms, dietary antigens, and environmental stressors and therefore represent important portals of pathogen entry and sites of local immune defense in fish [37,38]. In the gills, dietary low-molecular-weight chitosan was associated with higher transcript abundance of *il8*, *il10*, *lyz*, *c3*, *tgfb1*, and *zo1*, indicating activation of inflammatory signaling, regulatory cytokine responses, innate antibacterial defense, complement activity, and epithelial barrier-related protection. In the intestine, chitosan supplementation increased *il8*, *il10*, *tgfb1*, *zo1*, *ocln*, and *cldn3*, indicating transcriptional responses involving mucosal immune regulation and tight-junction-associated genes. However, epithelial integrity was not directly measured. These findings are in line with the known biological properties of low-molecular-weight chitosan, including improved solubility, interaction with mucosal surfaces, and immunomodulatory activity. They also agree with broader intestinal barrier studies showing that tight-junction complexes, including ZO-1, occludin, and claudin family proteins, regulate paracellular permeability and are central to the maintenance of epithelial barrier integrity in fish gills and intestine [39,40]. Maintenance of these structures contributes to mucosal homeostasis and may restrict the translocation of pathogens and harmful luminal components across the epithelium.

Although much of the detailed tight-junction literature comes from mammalian models, these mechanisms are relevant to fish mucosal health because epithelial barrier integrity is essential for reducing pathogen translocation and maintaining immune homeostasis. Chitosan oligosaccharide has been reported to protect intestinal barrier function by improving tight-junction-associated proteins and reducing inflammatory injury in animal models. Tao et al. reported that chitosan oligosaccharide alleviated LPS-induced intestinal injury and prevented downregulation of tight-junction proteins [41]. The present upregulation of *zo1*, *ocln*, and *cldn3* in Asian seabass intestine suggests that low-molecular-weight chitosan may be associated with mucosal barrier preparedness, but this interpretation requires confirmation using protein localization, permeability, or histological measurements, which could partly explain the improved resistance to *Photobacterium damselae* challenge. These transcriptional findings should be interpreted cautiously. Higher mRNA abundance indicates that the selected genes were transcriptionally responsive to dietary chitosan, but it does not demonstrate corresponding changes in protein abundance, post-translational regulation, enzyme activity, immune-cell function, or epithelial permeability. Therefore, the present results identify transcriptional patterns consistent with antioxidant, immune, and barrier-related responses rather than confirming activation of the corresponding biological pathways. Protein-level analyses and functional barrier assays will be required to verify these proposed mechanisms.

The survival and RPS results provide functional evidence that the observed immunological and antioxidant responses were associated with improved resistance to *P. damselae*, a bacterial pathogen previously isolated from diseased Asian seabass and associated with mortality in commercial production systems. Fish fed chitosan-supplemented diets showed improved survival after *P. damselae* challenge, with the highest RPS observed in the LwChitosan0.5 group. This agrees strongly with the earlier Asian seabass study by Ranjan et al., where dietary chitosan improved survival after *V. anguillarum* challenge [27]. Similar protective effects have also been reported in Nile tilapia fed chitosan nanoparticles and challenged with *Aeromonas veronii*, where treated groups showed lower mortality and improved immune status [19]. Therefore, the present study supports the broader conclusion that chitosan-based feed additives can enhance bacterial disease resistance in cultured fish, while adding new evidence that degraded low-molecular-weight chitosan produced by γ-irradiation and H_2_O_2_ is effective in Asian seabass.

The dose–response pattern was endpoint-specific rather than uniformly dose-dependent. LwChitosan0.25 produced the highest total serum IgM response, whereas LwChitosan1.0 produced the highest CAT activity. In contrast, LwChitosan0.5 showed the most consistent combined response across GSH, GPx activity, several tissue-specific transcriptional responses, and post-challenge protection. Therefore, 0.5% should not be interpreted as a universally optimal inclusion level. Rather, it represented the most consistently responsive concentration across the combined endpoints evaluated under the conditions of this four-week study. Differences among endpoints may reflect variation in their sensitivity, kinetics, and physiological regulation. A dedicated dose-optimization study incorporating additional inclusion levels and longer feeding periods is required before an optimal practical or commercial supplementation level can be established.

An important limitation of the present molecular analysis is that the qRT-PCR data represent transcriptional responses and do not necessarily indicate corresponding changes in protein abundance or biological activity. Changes in mRNA abundance may be modified by post-transcriptional regulation, translation efficiency, protein turnover, and post-translational regulation. Therefore, increased expression of antioxidant-, immune-, or barrier-related genes should not be interpreted as direct evidence of increased protein production or activation of the corresponding biological pathways. Although some transcriptional responses were accompanied by changes in serum antioxidant and immune measurements, the present design does not establish direct mechanistic relationships between these endpoints. Future studies incorporating proteomic or targeted protein analyses, enzyme activity measurements where appropriate, histopathology, epithelial permeability assays, and immune-cell characterization would provide stronger functional validation of the transcriptional findings. In addition, the four-week duration represents an important limitation of the present study. This period was sufficient to identify short-term serum, transcriptional, and post-challenge responses, but it may not have allowed small differences in somatic growth or feed utilization to accumulate to statistically detectable levels. Moreover, a response measured at the end of four weeks does not establish that antioxidant or immune effects will persist during longer production cycles. Longer feeding trials that include repeated sampling, grow-out performance, and post-supplementation follow-up are required to determine the duration, stability, and practical relevance of these responses.

Overall, the present findings indicate that low-molecular-weight chitosan produced by γ-irradiation and hydrogen peroxide depolymerization acts mainly as a health-promoting immunonutritional additive rather than a direct growth promoter during a short feeding period. Its beneficial effects were expressed through reduced oxidative damage, enhanced glutathione-based antioxidant defense, increased total IgM, broad activation of systemic and mucosal immune gene networks, improved tight-junction-related gene expression, and increased survival following bacterial challenge. Compared with native high-molecular-weight chitosan, the degraded low-molecular-weight form, especially at 0.5%, generally produced stronger and more consistent responses. These results support the potential application of 0.5% low-molecular-weight chitosan as a sustainable functional feed additive for improving health status and bacterial disease resilience in Asian seabass aquaculture.

## 4. Materials and Methods

### 4.1. Materials and Preparation of Synergistically Degraded Chitosan

Low-molecular-weight chitosan was produced through a combined oxidative and γ-irradiation process prior to its incorporation into experimental diets. Native chitosan was supplied by Bio21 Co., Ltd. (Chonburi, Thailand). According to the supplier’s certificate of analysis, the material had a degree of deacetylation exceeding 95% and a nominal molecular weight of approximately 500 kDa. The degree of deacetylation was not independently remeasured before or after the combined depolymerization treatment. Lactic acid (85%) was purchased from Ajax Finechem Pty Ltd. (Taren Point, NSW, Australia), while hydrogen peroxide (30%) was obtained from Merck KGaA (Darmstadt, Germany).

To prepare the degraded chitosan, 15 g of chitosan was dissolved in 400 mL of 1.5% (*w*/*v*) lactic acid with continuous magnetic stirring until complete dissolution was achieved. Hydrogen peroxide was subsequently introduced to obtain a final concentration of 0.25% (*w*/*v*). The solution was then irradiated using a ^60^Co gamma source at the Thailand Institute of Nuclear Technology (Public Organization), Headquarters, Thailand, at an absorbed dose of 10 kGy with a dose rate of 6.244 kGy h^−1^ under ambient conditions.

For comparison, native chitosan was prepared following the same dissolution procedure but without hydrogen peroxide addition or γ-irradiation, and served as the high-molecular-weight chitosan sample.

The molecular weight and molecular weight distribution of both chitosan samples were analyzed by aqueous gel permeation chromatography (GPC) using a Shimadzu HPLC system equipped with a refractive index detector and a Shodex SB-804 HQ column packed with polymeric polyether gel. Dried chitosan samples (20 mg) were dissolved in 10 mL of 2% (*w*/*v*) acetic acid to obtain a concentration of 2 mg mL^−1^ before analysis. Pullulan standards were employed for calibration, while 0.2 M sodium acetate served as the mobile phase at a flow rate of 0.5 mL min^−1^. The column temperature was maintained at 30 °C, and an injection volume of 20 μL was used for each sample.

The apparent viscosity of the chitosan solutions was determined at 25 °C using a HAAKE RheoStress rheometer (Thermo Scientific, Karlsruhe, Germany) under a constant shear rate of 100 s^−1^. Each sample was measured in quintuplicate.

Under the GPC conditions used in this study and relative to pullulan standards, the untreated preparation had an apparent average molecular weight of approximately 85 kDa, whereas the preparation subjected to 0.25% H_2_O_2_ and 10 kGy gamma irradiation had an apparent average molecular weight of approximately 10 kDa. Based on these physicochemical properties, together with their viscosity characteristics and previously reported antibacterial performance, both native and degraded chitosan were incorporated into Asian seabass feed formulations as functional dietary additives [18]. A graphical overview of the preparation, characterization, and dietary application of the chitosan formulations is provided in Supplementary Figure S1.

### 4.2. Effects of Synergistically Degraded Low-Molecular-Weight Chitosan on Health Promotion, Antioxidant Responses, and Disease Resistance in Asian Seabass (Lates calcarifer)

#### 4.2.1. Ethics Statement

All experimental procedures involving fish were conducted in accordance with the Ethical Principles and Guidelines for the Use of Animals for Scientific Purposes established by the National Research Council of Thailand. The experimental protocol was reviewed and approved by the Animal Ethics Committee of Kasetsart University, Thailand (Approval No. ACKU67-FIS-017; approved on 20 May 2024). All procedures were designed to minimize handling stress, discomfort, and unnecessary mortality throughout the experimental period.

#### 4.2.2. Animal Husbandry

Juvenile Asian seabass (*Lates calcarifer*) with an average initial body length of 10.0 ± 2.0 cm and body weight of 20.0 ± 3.0 g were obtained from a commercial hatchery in Thailand. Upon arrival, fish were transferred to fiberglass tanks containing continuously aerated brackish water and acclimated under laboratory conditions for 14 days before the feeding trial. During acclimation, water salinity was maintained at 5 ppt, temperature at 28 ± 2 °C, dissolved oxygen was maintained at ≥5 mg/L, and pH ranged from 7.0 to 8.0. During the acclimation period, fish were fed a commercial seabass diet containing approximately 42% crude protein and 9% crude lipid at 4–5% of body weight per day, divided into two daily feedings. Feed rations were adjusted according to apparent feed intake and fish biomass. Uneaten feed and fecal matter were removed daily by siphoning, and partial water exchange was performed regularly using dechlorinated brackish water adjusted to the same salinity.

Fish health was monitored daily throughout acclimation. Swimming behavior, feeding activity, external appearance, pigmentation, and clinical signs of disease were observed. Moribund or dead fish, if present, were removed immediately and recorded. Before the start of the experiment, a subset of fish was randomly examined to confirm the absence of visible abnormalities and overt bacterial infection. Only healthy and active fish were selected for the feeding trial.

#### 4.2.3. Experimental Design and Experimental Diet Preparation

Two chitosan preparations were used in this study: native non-degraded chitosan with an approximate molecular weight of 85 kDa and synergistically degraded low-molecular-weight chitosan with an approximate molecular weight of 10 kDa. The low-molecular-weight chitosan was produced by γ-irradiation at 10 kGy in combination with 0.25% (*w*/*v*) H_2_O_2_. These chitosan preparations were incorporated into seabass diets as functional feed additives to evaluate their potential roles in health promotion, antioxidant regulation, immune modulation, and bacterial disease resistance.

A 4-week feeding trial was conducted using a completely randomized design. Five dietary treatments were prepared, with each treatment assigned to four replicate fiberglass tanks. Juvenile Asian seabass were randomly distributed into tanks containing aerated brackish water. Each tank contained 30 fish, giving a total of 120 fish per treatment.

The experimental diets were arranged as follows:

1. Control: basal diet coated with sterile distilled water
2. HwChitosan1.0: basal diet supplemented with non-degraded high-molecular-weight chitosan at 1.0% (*w*/*w*)
3. LwChitosan0.25: basal diet supplemented with degraded low-molecular-weight chitosan at 0.25% (*w*/*w*)
4. LwChitosan0.5: basal diet supplemented with degraded low-molecular-weight chitosan at 0.5% (*w*/*w*)
5. LwChitosan1.0: basal diet supplemented with degraded low-molecular-weight chitosan at 1.0% (*w*/*w*)

The dietary levels were selected based on previous fish studies and our earlier evaluation of synergistically degraded chitosan [17,18]. A 1.0% inclusion level was selected for the untreated high-molecular-weight preparation because 10 g/kg dietary chitosan produced favorable immune and disease-resistance responses in Asian seabass. Low-molecular-weight chitosan was evaluated at 0.25%, 0.5%, and 1.0% to examine whether molecular-weight reduction could produce biological responses at lower dietary levels and to permit a direct comparison between the high- and low-molecular-weight preparations at the common 1.0% inclusion level. These levels were selected for biological screening and were not assumed a priori to represent optimal commercial doses.

A commercial extruded marine fish diet manufactured by Thai Union Feedmill Public Company Limited, Samut Sakhon, Thailand, under the PROFEED brand was used as the basal diet. The feed had a pellet diameter of approximately 2.0 mm and contained not less than 42% crude protein, according to the manufacturer’s specifications. The high-molecular-weight and low-molecular-weight chitosan coating solutions were prepared at concentrations sufficient to provide final dietary inclusion levels of 1.0% HwChitosan and 0.25%, 0.5%, or 1.0% LwChitosan on a weight-by-weight basis. Chitosan was dissolved in lactic acid, and each coating solution was prepared immediately before application. A total volume of 100 mL of coating solution per kilogram of feed was evenly sprayed onto the basal diet while the pellets were continuously mixed to ensure uniform distribution.

After coating, the experimental diets were spread in a single layer and air-dried at room temperature for 1 h until the surface moisture had completely evaporated. Fresh batches were prepared at regular intervals to minimize deterioration of the feed and loss of chitosan activity.

During the feeding trial, the amount of feed offered to each tank was recorded daily. Fish were fed at 3 to 5% of body weight per day, divided into two equal meals. Feeding allowances were adjusted weekly according to tank biomass and apparent feed consumption.

During the experiment, salinity was maintained at 5 ppt, water temperature at 28 ± 2 °C, dissolved oxygen at ≥ 5 mg/L, and pH between 7.0 and 8.0. Uneaten feed and feces were removed daily, and partial water exchange was conducted routinely. Fish survival, feeding response, swimming behavior, external appearance, and general health condition were recorded daily.

At the sampling time, two fish were randomly selected from each tank (8 fish/group). Fish were anesthetized with clove oil at 50 mg/L until loss of equilibrium. Blood samples were collected from the caudal vein using sterile syringes. Serum was separated according to the requirements of antioxidant enzyme and immune assays. Liver, head kidney, spleen, intestine, and gills were aseptically dissected and preserved for gene expression analysis.

#### 4.2.4. Growth Performance Analysis

Growth performance of Asian seabass (*Lates calcarifer*) was evaluated at the beginning and at the end of the 4-week feeding trial. At each sampling point, eight fish from each tank (4 tanks/group) were gently netted, anesthetized, blotted dry with tissue paper, and individually weighed using a digital balance. Initial body weight, final body weight, feed intake, and survival were recorded for each replicate tank. Mortality was checked daily throughout the experiment, and the number of surviving fish was recorded at the end of the trial. Growth performance and feed utilization were calculated using the following equations:

WG (g/fish) = final body weight − initial body weight

ADG (g/day) = (final body weight − initial body weight)/experimental period

SGR (%/day) = [(ln final body weight − ln initial body weight)/experimental period] × 100

FCR = total feed intake (g)/total weight gain (g)

Feed intake was calculated for each replicate tank from the total amount of feed offered after accounting for uneaten feed.

#### 4.2.5. Collection of Blood and Serum

Blood and serum samples were collected from Asian seabass (*Lates calcarifer*) at the end of the 4-week feeding trial. Two fish were randomly selected from each replicate tank (8 fish/group) and anesthetized with clove oil at 50 ppm until loss of equilibrium was observed. The body surface was gently wiped with sterile tissue to reduce external contamination before blood collection.

Whole blood was withdrawn from the caudal vein using a sterile 1.0 mL syringe fitted with a fine needle. For serum preparation, blood samples were transferred into sterile microcentrifuge tubes without anticoagulant and allowed to clot at room temperature for approximately 1 h. The clotted blood was then centrifuged at 7500× *g* for 10 min at 4 °C. The clear serum fraction was carefully collected and stored at −20 °C until analysis of humoral immune responses, oxidative stress markers, and antioxidant enzyme activities.

#### 4.2.6. qRT-PCR Analysis of Growth-, Antioxidant-, and Immune-Related Genes

At the end of the 4-week feeding trial, tissue samples including liver, head kidney, spleen, gills, and intestine were aseptically dissected and collected from Asian seabass (*Lates calcarifer*) for gene expression analysis. Two fish were randomly selected from each replicate tank (8 fish/group) and anesthetized as described above.

Total RNA was extracted from tissue samples using the RNeasy kit (Qiagen, Hilden, Germany), according to the manufacturer’s instructions. The concentration and purity of the extracted RNA were evaluated using a NanoDrop™ spectrophotometer (Thermo Fisher Scientific, Waltham, MA, USA). RNA samples with acceptable purity, based on A260/A280 and A260/A230 ratios, were selected for cDNA synthesis. First-strand cDNA was synthesized from 1 µg of total RNA using Maxime™ RT PreMix (iNtRON Biotechnology, Seongnam-si, Gyeonggi-do, Republic of Korea), following the manufacturer’s protocol. The synthesized cDNA was stored at −20 °C until quantitative real-time PCR analysis.

Quantitative real-time PCR was performed using the QuantiNova SYBR Green qPCR Kit (Qiagen, Hilden, Germany) with an AriaMx Real-Time PCR System (Agilent Technologies, Santa Clara, CA, USA). The amplification program consisted of an initial denaturation at 95 °C for 5 min, followed by 40 amplification cycles of denaturation at 95 °C for 2 s, annealing at 60 °C for 20 s, and extension at 72 °C for 30 s. Melting curve analysis was conducted at the end of each run to confirm the specificity of PCR amplification and the absence of non-specific products or primer dimers.

The expression levels of growth-, antioxidant-, and immune-related genes were normalized using three reference genes, including *actb*, *ef1a*, and *gapdh*. Relative gene expression was calculated using the 2^−ΔΔCt^ method. All primer pairs were validated for amplification efficiency and specificity before qRT-PCR analysis. The primer sequences of all target and reference genes are provided in Table 1.

#### 4.2.7. Measurement of Oxidative Stress Markers and Antioxidant Enzyme Activities in Serum

Oxidative stress markers and antioxidant enzyme activities were measured in serum samples collected from Asian seabass (*Lates calcarifer*) at the end of the 4-week feeding trial. Fish were randomly selected from each replicate tank and anesthetized before blood collection. Blood samples were withdrawn from the caudal vein using sterile syringes and transferred into sterile microcentrifuge tubes without anticoagulant.

The blood samples were allowed to clot at room temperature for approximately 1 h and then centrifuged at 7500× *g* for 10 min at 4 °C. The clear serum fraction was carefully collected without disturbing the clot and transferred into new sterile microcentrifuge tubes. Serum samples were stored at −20 °C until analysis.

The collected serum was used for the determination of oxidative stress markers and antioxidant enzyme activities, including malondialdehyde (MDA), reduced glutathione (GSH), nitric oxide (NO), glutathione reductase (GR), catalase (CAT), superoxide dismutase (SOD), glutathione peroxidase (GPx), and glutathione-S-transferase (GST). All assays were performed according to the respective standard protocols as follows:

(1) Thiobarbituric acid reactive substances (TBARS) assay

Lipid peroxidation was estimated by measuring malondialdehyde (MDA) levels using the TBARS method. Briefly, 0.1 mL of serum was mixed with 0.2 mL of thiobarbituric acid solution and 1.0 mL of trichloroacetic acid, followed by the addition of an equal volume of 0.85% saline. The mixture was heated at 100 °C for 30 min and cooled to room temperature. Then, 2.0 mL of distilled water was added, and the mixture was centrifuged at 3500 rpm for 10 min. The absorbance of the clear supernatant was measured at 532 nm against a blank. MDA concentration was calculated using a tetramethoxypropane standard curve and expressed as nmol MDA/mg protein.

(2) Reduced glutathione (GSH) content

Reduced glutathione levels were determined using a modified colorimetric method. Briefly, 1.0 mL of serum was mixed with 1.0 mL of 4% sulfosalicylic acid and incubated at 4 °C for 1 h to precipitate proteins. After centrifugation at 3500 rpm for 20 min at 4 °C, the supernatant was mixed with 100 mM phosphate buffer, pH 7.4, and 100 mM 5,5′-dithiobis-(2-nitrobenzoic acid), also known as DTNB. The formation of the yellow-colored product was measured at 412 nm. GSH concentration was calculated using a GSH standard curve and expressed as nmol GSH/mg protein.

(3) Nitric oxide (NO) determination

Nitric oxide production was estimated by measuring nitrite concentration using the Griess reaction. Equal volumes of serum and Griess reagent were mixed and incubated at room temperature. The absorbance of the resulting purple compound was measured at 546 nm. Nitrite concentration was calculated from a sodium nitrite standard curve and expressed as nmol nitrite/mg protein.

(4) Glutathione reductase (GR) activity

Glutathione reductase activity was assessed by monitoring NADPH oxidation. Briefly, 0.1 mL of serum was added to a reaction mixture containing 0.1 M phosphate buffer, pH 7.6, oxidized glutathione, and NADPH. The decrease in absorbance at 340 nm was recorded spectrophotometrically. GR activity was calculated using the extinction coefficient of NADPH and expressed as nmol NADPH oxidized/min/mg protein.

(5) Catalase (CAT) activity

Catalase activity was measured based on the decomposition of hydrogen peroxide. The reaction mixture consisted of 0.1 mL of serum, 2.5 mL of 50 mM phosphate buffer, pH 5.0, and 0.4 mL of 5.9 mM H_2_O_2_. The decrease in absorbance was recorded at 240 nm every 30 s for 2 min. CAT activity was calculated and expressed as U/mg protein.

(6) Superoxide dismutase (SOD) activity

Superoxide dismutase activity was determined by measuring the inhibition of nitroblue tetrazolium reduction. Briefly, 0.1 mL of serum was mixed with assay buffer containing 0.1 mM xanthine, 0.025 mM nitroblue tetrazolium, 0.1 mM EDTA, 60 mM sodium carbonate buffer, pH 10.2, and xanthine oxidase. The absorbance was measured at 560 nm. SOD activity was calculated using an SOD standard curve and expressed as U/mg protein.

(7) Glutathione peroxidase (GPx) activity

Glutathione peroxidase activity was measured by monitoring NADPH oxidation in the presence of glutathione reductase and reduced glutathione. The reaction mixture contained 0.1 M phosphate buffer, pH 7.4, 1 mM EDTA, 1 mM sodium azide, 1 U/mL glutathione reductase, 1 mM reduced glutathione, 0.2 mM NADPH, and 0.25 mM H_2_O_2_. The reaction was initiated by adding 0.1 mL of serum, and the decrease in absorbance at 340 nm was recorded continuously for 3 min. GPx activity was calculated using the extinction coefficient of NADPH and expressed as nmol NADPH oxidized/min/mg protein.

(8) Glutathione-S-transferase (GST) activity

Glutathione-S-transferase activity was determined using 1-chloro-2,4-dinitrobenzene, also known as CDNB, as the substrate. The reaction mixture contained 1.475 mL of 0.1 M phosphate buffer, pH 6.5, 0.2 mL of reduced glutathione, 0.025 mL of CDNB, and 0.3 mL of serum. The formation of the GSH-CDNB conjugate was monitored at 340 nm. GST activity was calculated and expressed as nmol GSH-CDNB conjugate formed/min/mg protein.

#### 4.2.8. Humoral Immune Response Assays

(1) Lysozyme activity

Serum lysozyme activity was measured using a turbidimetric method based on the ability of lysozyme to lyse *Micrococcus lysodeikticus*, following the principle described by Mörsky, P. (1983) [45] with minor modifications. Briefly, M. lysodeikticus powder (Sigma-Aldrich, Darmstadt, Germany) was freshly prepared in phosphate-buffered saline (PBS, pH 6.2) to obtain a final concentration of 0.2 mg/mL. The bacterial suspension was kept at room temperature for 1 h before use to ensure uniform dispersion. For the assay, 10 µL of serum was transferred into each well of a flat-bottom 96-well microplate, followed by the addition of 250 µL of the prepared *M. lysodeikticus* suspension. The mixture was incubated at room temperature, and the reduction in turbidity was monitored by measuring absorbance at 450 nm using a microplate spectrophotometer (iMark™ Microplate Absorbance Reader, BIO-RAD, Hercules, CA, USA). Absorbance was recorded at 0 and 5 min. A PBS blank was included as a negative control to correct for any non-enzymatic decrease in turbidity.

The rate of absorbance reduction was calculated as follows:

ΔA450/min = [(A0 − A5)/5]sample − [(A0 − A5)/5]negative control

Lysozyme activity was reported as units per milliliter (U/mL). One unit of lysozyme activity was defined as the enzyme activity causing a decrease in absorbance of 0.001 per minute under the assay conditions. The activity was calculated using the following formula:

Lysozyme activity (U/mL) = (ΔA450/min × Vtotal)/(0.001 × Vsample) × DF

where ΔA450/min represents the reduction in absorbance at 450 nm per minute, Vtotal refers to the total reaction volume in the well (mL), Vsample indicates the volume of serum used in the assay (mL), and DF represents the dilution factor of the serum sample.

(2) Quantification of Total Immunoglobulin M (IgM) by Indirect ELISA

Serum total IgM levels were measured by indirect ELISA following the method of Uchuwittayakul et al. (2025) [46], with minor modifications. Briefly, a flat-bottom 96-well microplate was pre-coated with bicarbonate buffer (pH 9.6) and incubated for 2 h at room temperature or overnight at 4 °C to facilitate binding to the plate surface. After removal of the coating solution, the diluted serum of 1:10 in coating buffer was added to each well and incubated overnight at 4 °C to allow serum immunoglobulins to adsorb to the plate surface. Following incubation, the plate was washed three times with PBST, consisting of PBS containing 0.05% Tween-20 (*v*/*v*, pH 7.4), using an automated microplate washer (Bio-Rad ImmunoWash 1575, Marnes-la-Coquette, France). Non-specific binding sites were blocked by adding 100 µL/well of blocking buffer (VisualProtein BlockPRO™, Taipei, Taiwan), followed by incubation for 2 h at room temperature. The plate was then washed three times with PBST. Subsequently, 100 µL/well of anti-seabass IgM rabbit monoclonal antibody, diluted 1:2000 in blocking buffer, was added and incubated for 2 h at 4 °C. After three washes with PBST, 100 µL/well of HRP-conjugated anti-rabbit IgG secondary antibody, diluted 1:5000 in blocking buffer, was added and incubated for 1 h at room temperature. The plate was washed six times with PBST to reduce background signal.

Color development was performed by adding 100 µL/well of TMB One-Component HRP Microwell Substrate and incubating for 10 min at room temperature. The enzymatic reaction was stopped by adding 100 µL/well of 0.5 M sulfuric acid (H_2_SO_4_). Absorbance was then recorded at 450 nm using a spectrophotometer.

#### 4.2.9. Disease Resistance and Relative Percent Survival After *Photobacterium damselae* Challenge

*P. damselae* AAHM-PD2413, originally isolated from diseased Asian seabass, was identified by 16S rRNA gene sequencing and maintained at the Center of Excellence in Aquatic Animal Health Management (CE AAHM), Faculty of Fisheries, Kasetsart University, Bangkok, Thailand. For preparation of the bacterial challenge suspension, the isolate was cultured in tryptic soy broth supplemented with sterile marine water adjusted to 10 ppt and incubated at 30–32 °C for 24 h. Bacterial cells were harvested by centrifugation at 500× *g* for 10 min and washed twice with sterile 1.0% NaCl solution (pH 7.4). The bacterial concentration was estimated spectrophotometrically at 600 nm using a previously established calibration curve, in which an optical density of 1.0 corresponded to approximately 1 × 10^8^ CFU/mL. The suspension was subsequently adjusted to the required concentration for the *P. damselae* challenge.

After the 4-week feeding trial, 40 fish from each dietary group, consisting of 10 fish per replicate, were randomly selected for the bacterial challenge test. Fish were maintained in 250 L fiberglass tanks under the same water-quality conditions used during the feeding trial. For the challenge, each fish was intraperitoneally injected with 100 µL of *P. damselae* suspension at a concentration of 1 × 10^6^ CFU/mL, corresponding to 1 × 10^5^ CFU/fish. The challenge dose was selected based on a preliminary LD_50_ test in Asian seabass. Following bacterial challenge, fish were observed daily for 14 days. Mortality was recorded every day, and dead fish were removed immediately from the tanks. Clinical signs and general behavior were monitored at 12 h intervals throughout the challenge period, and the causative pathogen was confirmed in dead fish by bacterial re-isolation. At the end of the observation period, cumulative mortality was calculated for each group, and relative percent survival (RPS) was determined using the following equation:

RPS (%) = [1 − (mortality rate of treatment group/mortality rate of control group)] × 100

#### 4.2.10. Statistical and Data Analysis

The tank was considered the independent experimental unit. Growth performance, feed utilization, and survival during the feeding trial were analyzed using tank-level observations (*n* = 4 tanks per treatment). For serum biochemical, humoral immune, and gene-expression analyses, two fish were randomly sampled from each of the four replicate tanks, providing eight individual biological samples per treatment (*n* = 8 fish). Each fish was analyzed separately, without pooling. Because the fish were nested within tanks, tank was included as a random effect in the statistical model to account for the non-independence of fish originating from the same tank. Serum biochemical, humoral immune, and gene-expression data were analyzed using a mixed-effects model, with dietary treatment as a fixed effect and tank as a random effect. The eight individually analyzed fish per treatment were treated as biological samples nested within four replicate tanks.

Normality of model residuals and homogeneity of variance were assessed using the Shapiro–Wilk and Brown–Forsythe tests, respectively. Data meeting parametric assumptions were analyzed by one-way ANOVA followed by Tukey’s HSD multiple-comparison test at *p* < 0.05. Exact *p*-values are reported where available.

The cumulative survival analysis of fish challenged with *P. damselae* was calculated using the Kaplan–Meier method. Cumulative survival plots were generated with GraphPad Prism version 10.1.2 (GraphPad Software, San Diego, CA, USA). Statistical significance between the control and treatment groups was denoted by different lowercase letters above the bars indicate significant differences among treatments at *p* < 0.05.

## 5. Conclusions

This study showed that dietary chitosan influenced selected antioxidant, humoral immune, transcriptional, and post-challenge outcomes in Asian seabass during a four-week laboratory trial. Neither high-molecular-weight nor low-molecular-weight chitosan significantly affected growth or feed utilization. Compared with the Control and 1.0% high-molecular-weight chitosan treatments, the low-molecular-weight preparations produced broader responses across several health-related endpoints. Among the levels evaluated, 0.5% low-molecular-weight chitosan produced the most consistent combined response across several antioxidant, transcriptional, and post-challenge endpoints. However, individual responses were endpoint-specific, with total serum IgM and CAT activity showing their highest responses at 0.25% and 1.0%, respectively. Therefore, the present findings do not establish 0.5% as a universally optimal dose, and further dose–response studies are required before a practical inclusion recommendation can be made. The gene-expression findings indicate transcriptional responsiveness but do not confirm corresponding changes in protein abundance or pathway activity. In addition, the short trial duration, incomplete post-treatment structural characterization, absence of histopathological and functional barrier measurements, and limited number of independent tanks restrict broader interpretation. Thus, low-molecular-weight chitosan produced by combined gamma irradiation and hydrogen peroxide treatment is a promising candidate functional feed additive, but longer dose–response studies and validation under commercial farming conditions are required before a practical inclusion recommendation can be established.

## Figures and Tables

**Table 1:** Primers used for gene expression analysis in this study.

Gene Group	Gene Symbol	Gene Name	Primer 5′→3′	Tm (°C)	Target Organ	Primer Source/Reference
Antioxidant/redox	*inos*	*inducible Nitric Oxide Synthase*	F: CTGCAGGGCTCTCTGGTTTTA R: GCACATCCTCAGCGGTACAT	60	Liver	XM_018660027.2
	*ampk*	*AMP-activated protein kinase*	F: TCTGGGATGTAGGGAGTGGA R: GACTCTGACCCAAGGACTGC	60	Liver	XM_018670205.2
	*sod1*	*superoxide dismutase 1*	F: TGCGGCCAGACTATGTTAAG R: GTATCAGTGTTGGTGGTCAGT	60	Liver	XM_018675982.1
	*cat*	*catalase*	F: GAGTCTGCATCAGGTGTCTTT R: CAAACTGGTTAATGCTGATGGG	60	Liver	XM_018675907.1
	*gpx1*	*glutathione peroxidase 1*	F: GGCTGGGAGTGTTGAAGAG R: TTGCTGGAGTAACGAGAGTG	60	Liver	XM_018686718.2
	*igf1*	*insulin-like growth factor 1*	F: ACGCTGCAGTTTGTATGTGG R: CCTTAGTCTTGGGAGGTGCA	60	Liver	[42]
	*gh*	*growth hormone*	F: ACTGAAGACTGGAATCCTGCTG R: CACCTTGTGCATGTCCTTCTTG	60	Liver	XM_018661387.2
Immune-related genes	*mx*	*myxovirus resistance protein 1*	F: GAGGTCATCCACCTGAAGAAGG R: TGAGCTTCTCAGCCAGTTTAGG	60	Head kidney	[43]
	*ifng*	*interferon gamma*	F: TACCAGGAGCAGGACAAGC R: TCGTCAGGCAGCGAACTT	60	Head kidney	[43]
	*il8*	*interleukin 8*	F: GCATCATCAAGGAGAGAAAGCC R: GTGTCTGCTCAGCTTGTTTCTT	60	Head kidney, Gill, Intestine	[44]
	*tnfb*	*tumor necrosis factor beta*	F: AGGACTCCGCTGAGAAAAC R: TGAACGATGCCTGGCTGTA	60	Head kidney	XM_018699809
	*il10*	*interleukin 10*	F: GCTAGATCAGACCGTCGAAGAC R: TGACATCACTCTTGAGCTCGTC	60	Head kidney, Spleen, Gill, Intestine	[42]
	*lyz*	*lysozyme*	F: TGCATCACACACCATGGCAA R: CATCCACGTTGTCATAGGAG	60	Head kidney, Gill	[42]
	*c3*	*complement component 3*	F: CATCACCAAAGAAATGCTGCCA R: CTCATAAGACGGAGCAGGTCTC	60	Head kidney, Spleen, Gill	[42]
	*dcst*	*Ddendritic cell-specific transcript*	F: AAGACAGTAGACCTCTCCCACA R: CAAACAGGGGAAGGACTGAGAG	60	Head kidney	[43]
	*mhc2*	*major histocompatibility complex class II*	F: TTCCTACCTCCCTGATCTACCC R: CTGAAGTCGCTGTTGGAGTAGT	60	Head kidney, Spleen	[43]
	*cd4*	*CD4 molecule*	F: AGTGCAATGGATTGGGGTAGATAA R: GTTGCAGGCTCTGTAACTTTGATT	60	Spleen	[43]
	*tgfb1*	*transforming growth factor beta 1*	F: TACCTCGCTTCCCGTTTC R: CTGCTCATCCTCAGTCCCTC	60	Spleen, Gill, Intestine	XM_018665504
Stress response	*hsp70*	*heat shock protein 70*	F: CTGGAGTCCTACGCTTTCAA R: CTTGCTGATGATGGGGTTAC	60	Spleen	HQ646109
Tight junction	*zo1*	*zonula occludens-1*	F: TCCGTATTGGCAAGAACCATCA R: TCTTCCGCAAATTCCTCTTGGA	60	Gill, Intestine	XM_051073853.1
	*ocln*	*occludin*	F: CAGGAGTGTTCGTCAACCAGTA R: CGACAACCTTCACTCTCTTCCA	60	Intestine	XM_018697365.2
	*cldn3*	*claudin3*	F: GATCCTTGGGGTCATGATCTCC R: TGATCAGGAGAGGGCTGTAGAA	60	Intestine	XM_018692623.2
Reference gene	*actb*	*beta-actin*	F: TACCACCGGTATCGTCATGGA R: CCACGCTCTGTCAGGATCTTC	60	All organs	[42]
	*ef* *1* *a*	*elongation factor 1 alpha*	F: GTTGCCTTTGTCCCCATCTC R: CTTCCAGCAGTGTGGTTCCA	60	All organs	[42]
	*gapdh*	*glyceraldehyde-* *3-* *phosphate dehydrogenase*	F: CGCTTCCTGCACAACCAACT R: GTGGCAGTGATGGCATGAAC	60	All organs	[42]

## Data Availability

The original contributions presented in this study are included in the article/Supplementary Materials. Further inquiries can be directed to the corresponding author.

## Supplementary Materials

The following supporting information can be downloaded at: https://www.mdpi.com/article/10.3390/ijms27177772/s1.
