# Supplementary material for: Low-Molecular-Weight Chitosan Produced by Gamma Irradiation and Hydrogen Peroxide Promotes Immune Readiness, Antioxidant Responses, and Survival in Photobacterium damselae-Challenged Asian Seabass (Lates calcarifer)
Source: Int J Mol Sci. 2026 Aug 30;27(17):7772. doi: 10.3390/ijms27177772 (PMC13566582; doi:10.3390/ijms27177772)
Supplement: Supplementary file 1 [file ijms-27-07772-s001.zip › ijms-4463897-supplementary.pdf]

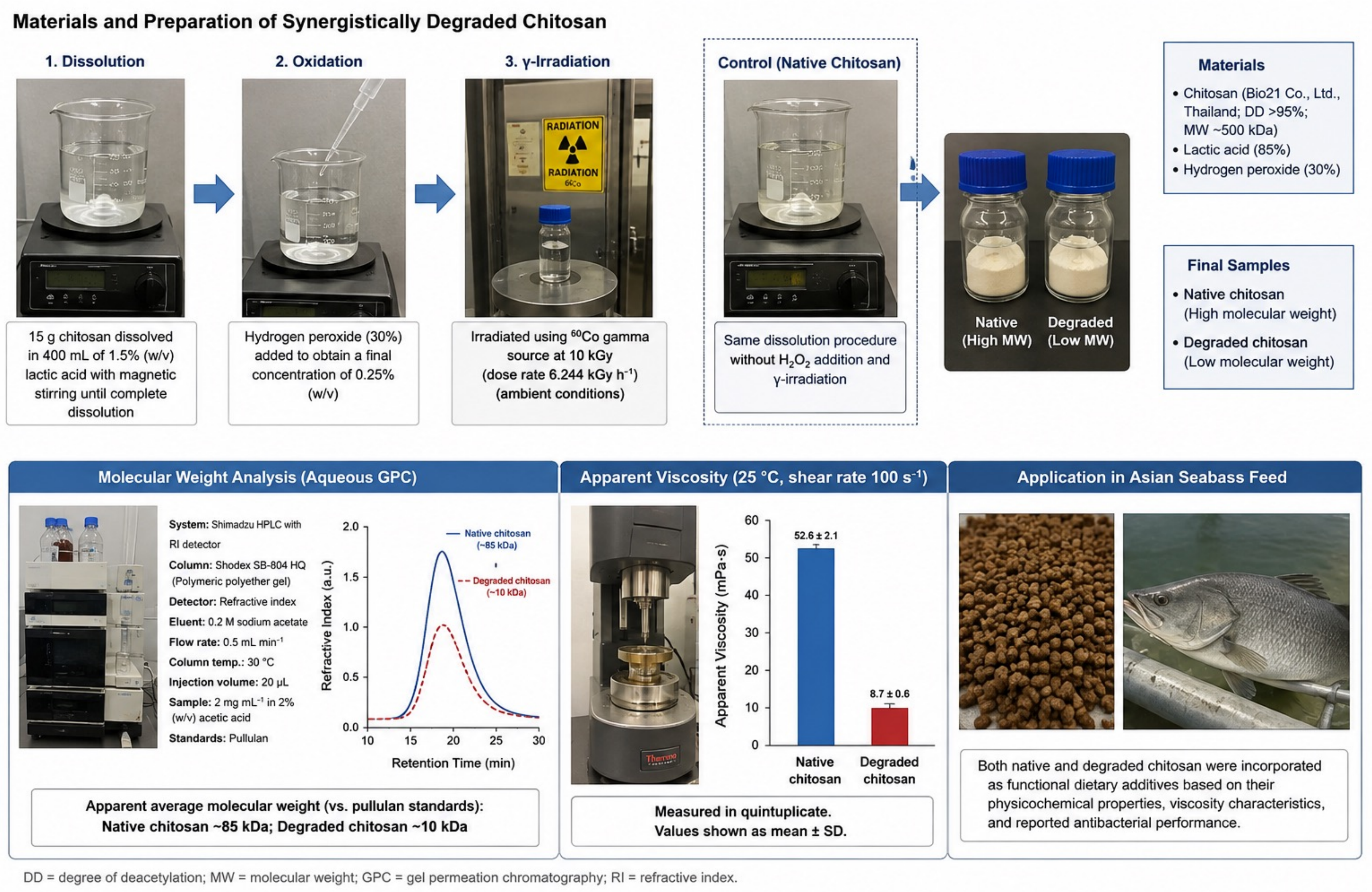

Figure S1. Graphical overview of the preparation, physicochemical characterization, and dietary application of synergistically degraded low-molecular-weight chitosan. Native chitosan was dissolved in 1.5% (w/v) lactic acid, supplemented with H_2O_2 to a final concentration of 0.25% (w/v), and exposed to 10 kGy gamma irradiation. The untreated and degraded preparations were characterized by GPC and apparent-viscosity analysis before incorporation into the experimental diets.

Declaration: Graphical elements were generated using OpenAI's image-generation tool and subsequently reviewed and edited by the authors to accurately reflect the experimental procedures. The images are illustrative and do not represent original experimental photographs or analytical data.
